# Supplementary material for: Etiology of oncogenic fusions in 5,190 childhood cancers and its clinical and therapeutic implication
Source: Nat Commun. 2023 Apr 5;14:1739. doi: 10.1038/s41467-023-37438-4 (PMC10076316; doi:10.1038/s41467-023-37438-4)
Supplement: Supplementary file 1 — Supplementary Figure [file 41467_2023_37438_MOESM1_ESM.pdf]

**Supplementary Fig. 1 Method.** (**a**) Determining fusion versioning. We used 4 methods (Fusion-catcher, STAR-Fusion, Arriba, Cicero) on each RNAseq sample to detect oncogenic fusions. 3’ end of exons from N’ genes and 5’ end of exons from C’ genes are extracted to build a junction library for all candidate in-frame fusions, which is in turn used to extract reads containing such junctions from the RNAseq bam and to obtain read counts. Among junctions with >0 read supports, the most downstream exon from N’ gene and the most upstream exon from C’ gene are used to define the fusion version. (**b**). Determining neo-splicing. With DNA breakpoint, we first reconstruct the DNA contig that can include non-template insertions (black), then determine all candidate acceptors (AG) and donors (GT) followed by *in silico* enumerating all potential splicing combinations by following intron-AG-exon-GT-intron structures. RNAseq reads were then aligned to all such combinations to identify the best mapping(s). Candidate splice sites corresponding to splicing combinations with positive read supports are considered functional.

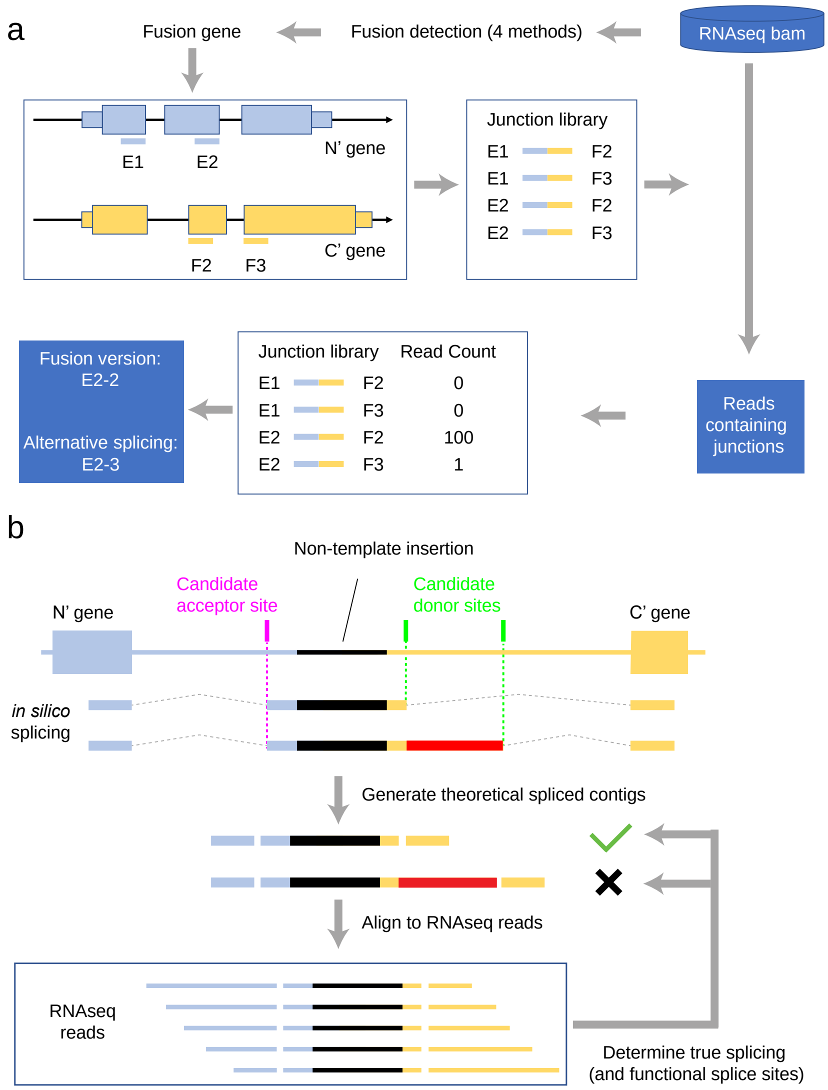

**Supplementary Fig. 2 Recurrent (n≥3) oncogenic fusions identified in 5,190 childhood cancers.** For each gene pair, coding exons (thick boxes) are colored white (frame 0), gray (frame 1), and black (frame 2). Intronic length is indicated by numbers. Gray lines indicate theoretically in-frame fusions. Red lines indicate in-frame fusions observed in patients and its width indicate patient prevalence. RefSeq identifiers are indicated for each involving gene. Fusions involving neo-splicing or chimeric exons were not included (see paper). Source data are provided in sheet Supplementary Fig.2 in Source Data file.

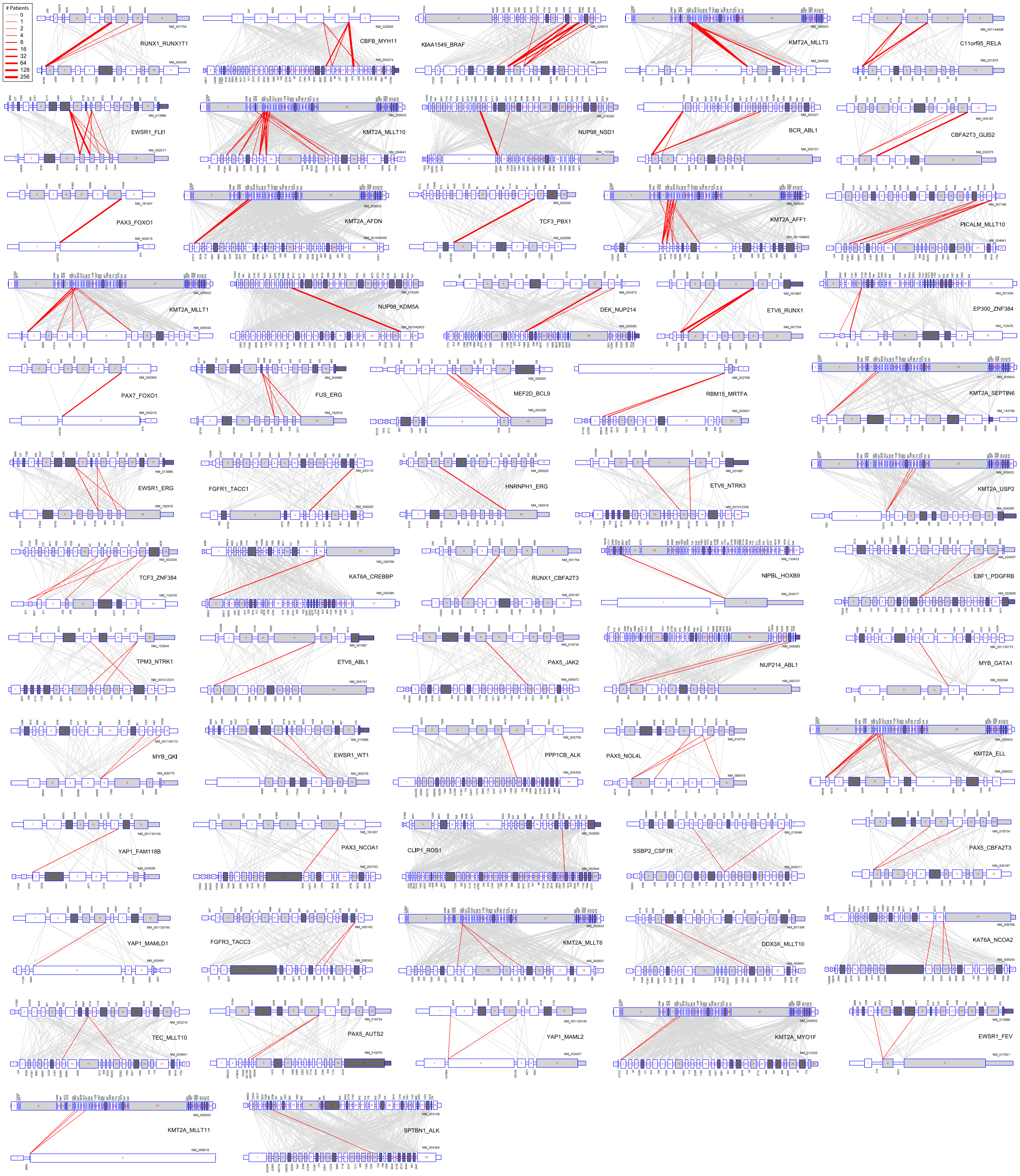

**Supplementary Fig. 3 Impact of gene length on prevalence of oncogenic fusions.** (**a**). DNA breakpoints can be detected from RNAseq data (see raw data in **Supplementary Data 8**). Shown in y-axis is the number of DNA breakpoints as a function of distance between DNA breakpoints detected from RNA and DNA sequencing data (n=43 samples with both data available) shown in x-axis (in base pairs). Note that each sample (oncogenic fusion) has two DNA breakpoints (totaling 86 breakpoints), one from N' gene and another from C' gene. Majority of detections from RNA data perfectly match that detected from DNA data. DNA breakpoints are uniformly distributed in *ETV6-RUNX1* (**b**), *NUP98-NSD1* (**c**), and demonstrated clustered distribution in promoter region of *RUNX1T1* gene for oncogenic fusion *RUNX1-RUNX1T1* (**d**), in addition to those shown in **Fig. 2j-i**. Also shown are linear regression between gene length and patient prevalence for fusions observed in leukemia (**e**), brain tumor (**f**), solid tumor (**g**) and all childhood cancer (**h**). In panels **b-d**, Bonferroni correction (Q values) is applied to the raw P values (two-sided Kolmogorov-Smirnov test) to account for multiple testing. For *KMT2A* (**i**), *ETV6* (**j**), and *PAX5* (**k**), there are sufficient (n≥5) fusion partner genes to study the association between patient prevalence and length of the partner genes. Only *KMT2A* partner genes resulted a significant association with R-squared of 0.86. Similar analysis was performed by only using the length of involved introns for leukemia (**l**), brain (**m**), solid (**n**), or all cancer types (**o**), as well as for partner genes for *KMT2A* (**p**), *ETV6* (**q**), and *PAX5* (**r**). Only *KMT2A* partner genes demonstrate significant association between intron length and patient prevalence (R-squared of 0.84). Removing *KMT2A* fusions from panels e and l resulted in insignificant P values (P=0.22; n=22; **Supplementary Fig. 4**). Linear model, P value and R-squared value are indicated for panels **e-r**. Source data are provided accordingly as sheet Supplementary Fig.3a-d and Supplementary Fig.3e-r in Source Data file.

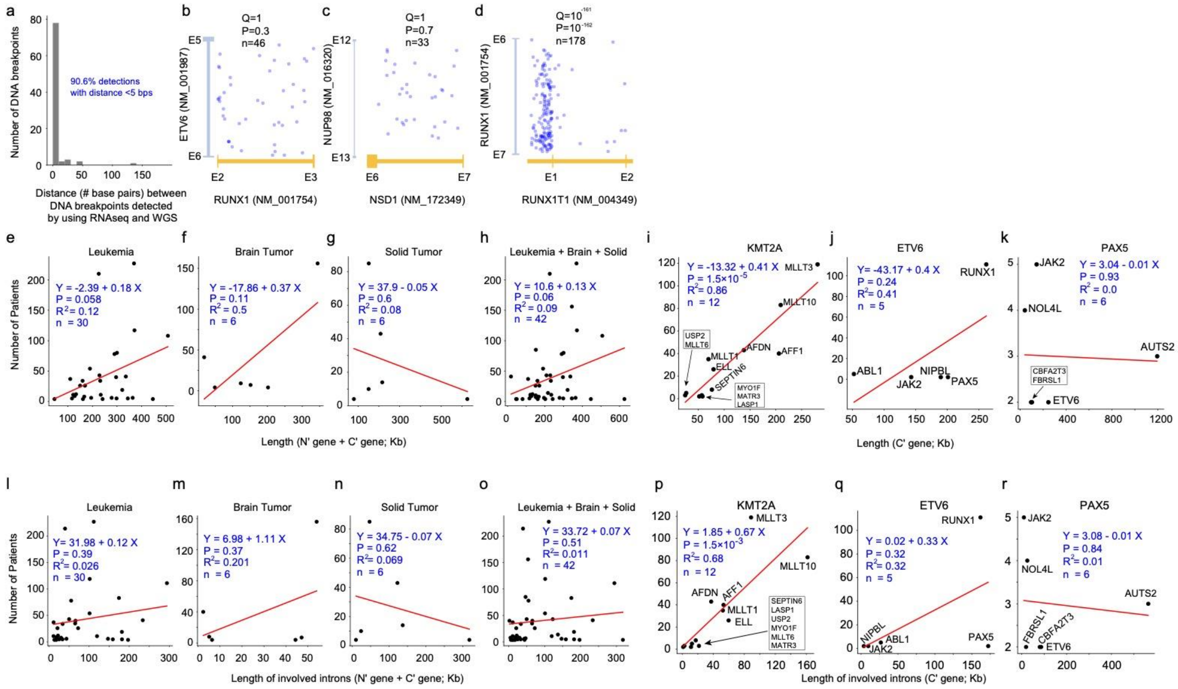

**Supplementary Fig. 4 Poor association of prevalence of oncogenic fusions (y-axis) with gene length (x-axis) in leukemia when *KMT2A* fusions were excluded**. Source data are provided in sheet Supplementary Fig.4 in Source Data file.

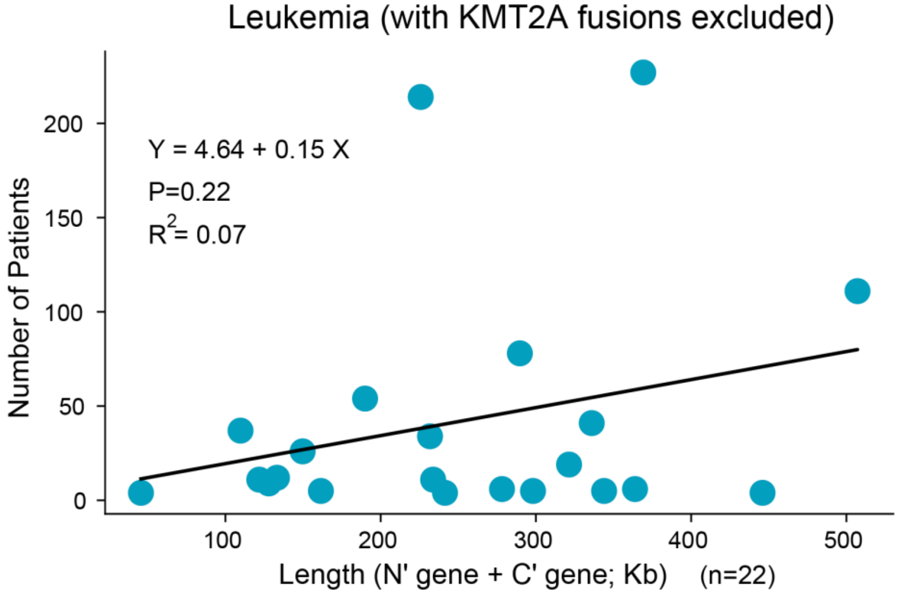

**Supplementary Fig. 5 Expression of childhood oncogenic fusions in GTEx samples.** Expression dominance score (EDS) of oncogenic fusions in all GTEx samples (**a**), in GTEx samples by matching relevant tissue types for the cancer types of the oncogenic fusions (**b**), where absent of green bars indicate lack of matching normal specimens in GTEx. Asterisks indicates Q value <0.01 (one-sided Wilcox rank sum test after Bonferroni correction for multiple testing, n=32). In boxplot the lower, center and upper limits indicate 25th, 50th, and 75th percentile, respectively. Whisker is defined using 1.5 IQR (**a-b**). Pattern of expression dominance is clearly observed in panel b for *TCF3-PBX1* fusion where *PBX1* is lowly or not expressed in normal blood, consistent with the observation in B-ALL (**Fig. 3b**). On the other hand, *FOXO1* appears to have higher expression than *PAX3*/*PAX7* in normal muscle/soft tissues, consistent with the observation in rhabdomyosarcoma, a type of sarcoma made up of cells that normally develop into skeletal muscles (**Fig. 3b**). Note the lack of normal myeloid specimens in GTEx cohort for AML fusions including *KMT2A*-rearrangements, *RUNX1-RUNX1T1*, and *CBFB-MYH11* fusions etc. Dotted horizontal red lines indicate 95% confidence interval of EDS scores determined in fusion positive samples. (**c**) Expression level of C' genes (gene name in red; measured as fragments per kilobase of exon per million mapped fragments or FPKM) of oncogenic fusions categorized as promoter-hijacking-like in **Fig. 3b**. Tumor samples are grouped into fusion positive and fusion negative groups. Also indicated are one-sided Wilcox rank sum test P values with false discover rate control (Q values). Dotted vertical red lines indicate median. Source data are provided accordingly as sheet Supplementary Fig.5a-b and Supplementary Fig.5c in Source Data file.

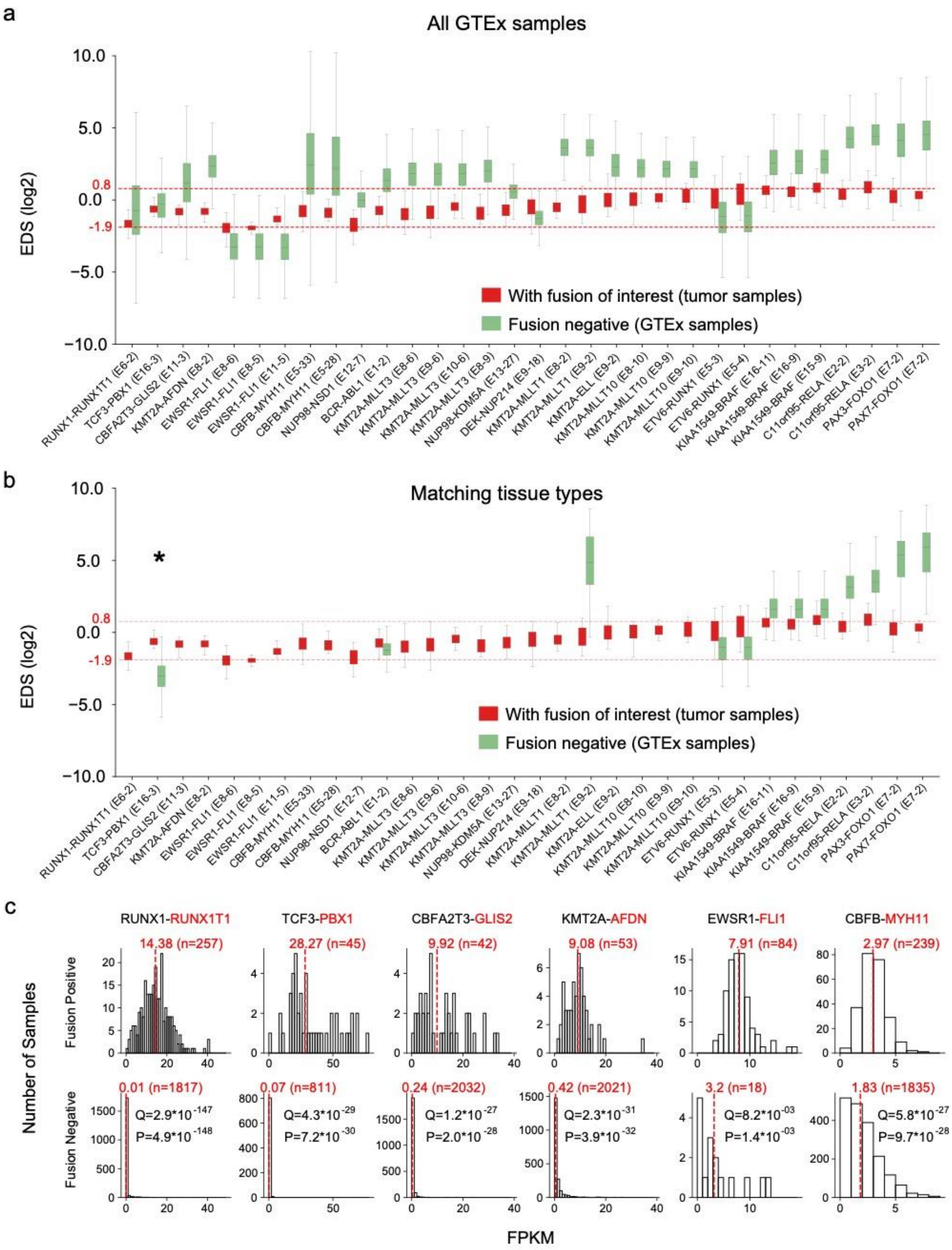

a
All GTEx samples
EDS (log2)
10.0
5.0
0.8
0.0
-1.9
-5.0
−10.0
With fusion of interest (tumor samples)
Fusion negative (GTEx samples)
RUNX1-RUNX1T1 (E6-2)
TCF3-PBX1 (E16-3)
CBFA2T3-GLIS2 (E11-3)
KMT2A-AFDN (E8-2)
EWSR1-FLI1 (E8-6)
EWSR1-FLI1 (E8-5)
EWSR1-FLI1 (E11-5)
CBFB-MYH11 (E5-33)
CBFB-MYH11 (E5-28)
NUP98-NSD1 (E12-7)
BCR-ABL1 (E1-2)
KMT2A-MLLT3 (E8-6)
KMT2A-MLLT3 (E9-6)
KMT2A-MLLT3 (E10-6)
KMT2A-MLLT3 (E8-9)
NUP98-KDM5A (E13-27)
DEK-NUP214 (E9-18)
KMT2A-MLLT1 (E8-2)
KMT2A-MLLT1 (E9-2)
KMT2A-ELL (E9-2)
KMT2A-MLLT10 (E8-10)
KMT2A-MLLT10 (E9-9)
KMT2A-MLLT10 (E9-10)
ETV6-RUNX1 (E5-3)
ETV6-RUNX1 (E5-4)
KIAA1549-BRAF (E16-11)
KIAA1549-BRAF (E16-9)
KIAA1549-BRAF (E15-9)
C11orf95-RELA (E2-2)
C11orf95-RELA (E3-2)
PAX3-FOXO1 (E7-2)
PAX7-FOXO1 (E7-2)
b
Matching tissue types
*
c
RUNX1-RUNX1T1
TCF3-PBX1
CBFA2T3-GLIS2
KMT2A-AFDN
EWSR1-FLI1
CBFB-MYH11
Number of Samples
Fusion Positive
Fusion Negative
14.38 (n=257)
28.27 (n=45)
9.92 (n=42)
9.08 (n=53)
7.91 (n=84)
2.97 (n=239)
0.01 (n=1817)
Q=2.9*10^-147
P=4.9*10^-148
0.07 (n=811)
Q=4.3*10^-29
P=7.2*10^-30
0.24 (n=2032)
Q=1.2*10^-27
P=2.0*10^-28
0.42 (n=2021)
Q=2.3*10^-31
P=3.9*10^-32
3.2 (n=18)
Q=8.2*10^-03
P=1.4*10^-03
1.83 (n=1835)
Q=5.8*10^-27
P=9.7*10^-28
FPKM

**Supplementary Fig. 6 Calculating splicing dominance score for wildtype genes and alternative splicing of oncogenic fusions in GTEx samples.** (**a**) Reads supporting all possible exon junctions are extracted and summarized to read counts for all splicing patterns (a, b, d, e, Y1-Y5). Similar as **Fig. 5a**, read count of expected splicing (Y1) and of all splicing patterns (Y2-Y5) spanning the intron of interest (blue) are used to define the splicing dominance score (SDS) for wildtype genes. (**b**) Alternative splicing of wildtype genes in all GTEx RNAseq samples. Splicing dominance score (SDS) of oncogenic fusions is calculated from its corresponding tumors. In boxplot the lower, center and upper limits indicate 25th, 50th, and 75th percentile, respectively. Whisker is defined using 1.5 IQR (a-b). (**c**) Similar to panel b, where the samples were selected to match the tissue type for given fusions (see **Supplementary Data 10**). For *KMT2A-AFDN*, *NUP98-NSD1*, *KMT2A-MLLT10*, and *KMT2A-MLLT3*, there are no GTEx samples with matched tissue type (myeloid), therefore the wildtype gene columns (light blue and orange) are left blank. Samples from non-matching tissues (panel a) masked the pattern observed in panel b, indicating the importance of tissue type matching. Concordant with tumor data in **Fig. 4b**, alternative splicing in *ETV6-RUNX1* is explained by alternative splicing in wildtype *RUNX1* observed in non-cancerous B cells from GTEx. Source data are provided in sheet Supplementary Fig.6b-c in Source Data file.

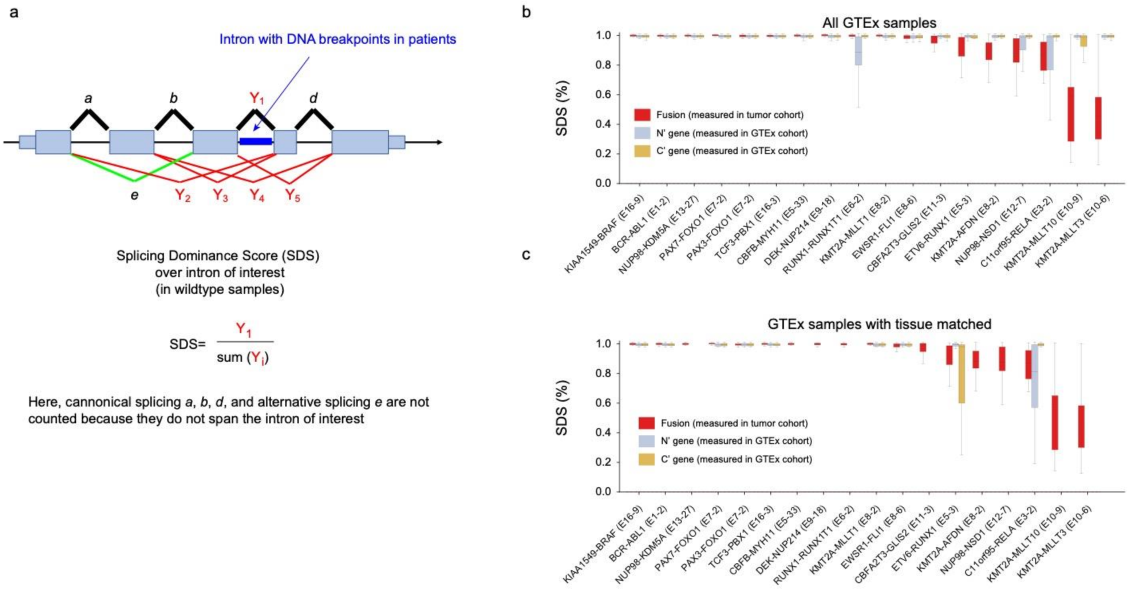

**Supplementary Fig. 7 Neo-splicing events**. Shown are brain tumor (**a**), leukemia (**b**), solid tumor (**c**), and cell line models (**d**). Each panel represents a tumor (with sample name indicated) with oncogenic fusion (blue: N' gene, orange: C' gene) detected. Exon/intron structures of wildtype N' gene (top) and C' gene (bottom) surrounding DNA breakpoints (linked by dashed black lines) are illustrated. Local DNA sequences encompassing the DNA breakpoints are illustrated in the middle, with sources colored accordingly (blue shading: from N' gene; orange shading: from C' gene; black shading: non-template insertion). Function of these local DNA sequences are further indicated by boxes: thin boxes: introns, thick boxes: exons. The rearrangement can create novel splice acceptor (AG) or donor (GT). For example, *MN1-PATZ1* in brain tumor SJHGG030895 has a novel splice acceptor, while *C11orf95-NCOA2* in brain tumor SJEPD031787 has a novel splice donor. Certain tumors can have alternative splicing around the neo-splice sites. For example, *C11orf95-NCOA2* in brain tumor SJEPD030512 has 3 splicing patterns, α, β, and ε. Interestingly, β and ε differed by 3 base pairs due to a local motif G<u>GT</u>G<u>GT</u> generated by the rearrangement that can create two splicing donors highlighted by underlined GT. (**e**) Exon 16 of *TCF3* and exon 4 of *HLF* have incompatible translation frames, therefore a "conventional fusion" connecting these two exons will not be functional, so that a neo splicing event is obligatory to overcome the translation problem. This explains the enrichment of neo-splicing events in *TCF3-HLF* positive tumors. Neo splicing cases (such as SJAMLM7010953, PAWWJN and SJAML016571; see **Supplementary Data 4**) with incomplete DNA breakpoint information were not shown in this figure. Source data are provided in sheet Supplementary Fig.7 in Source Data file.

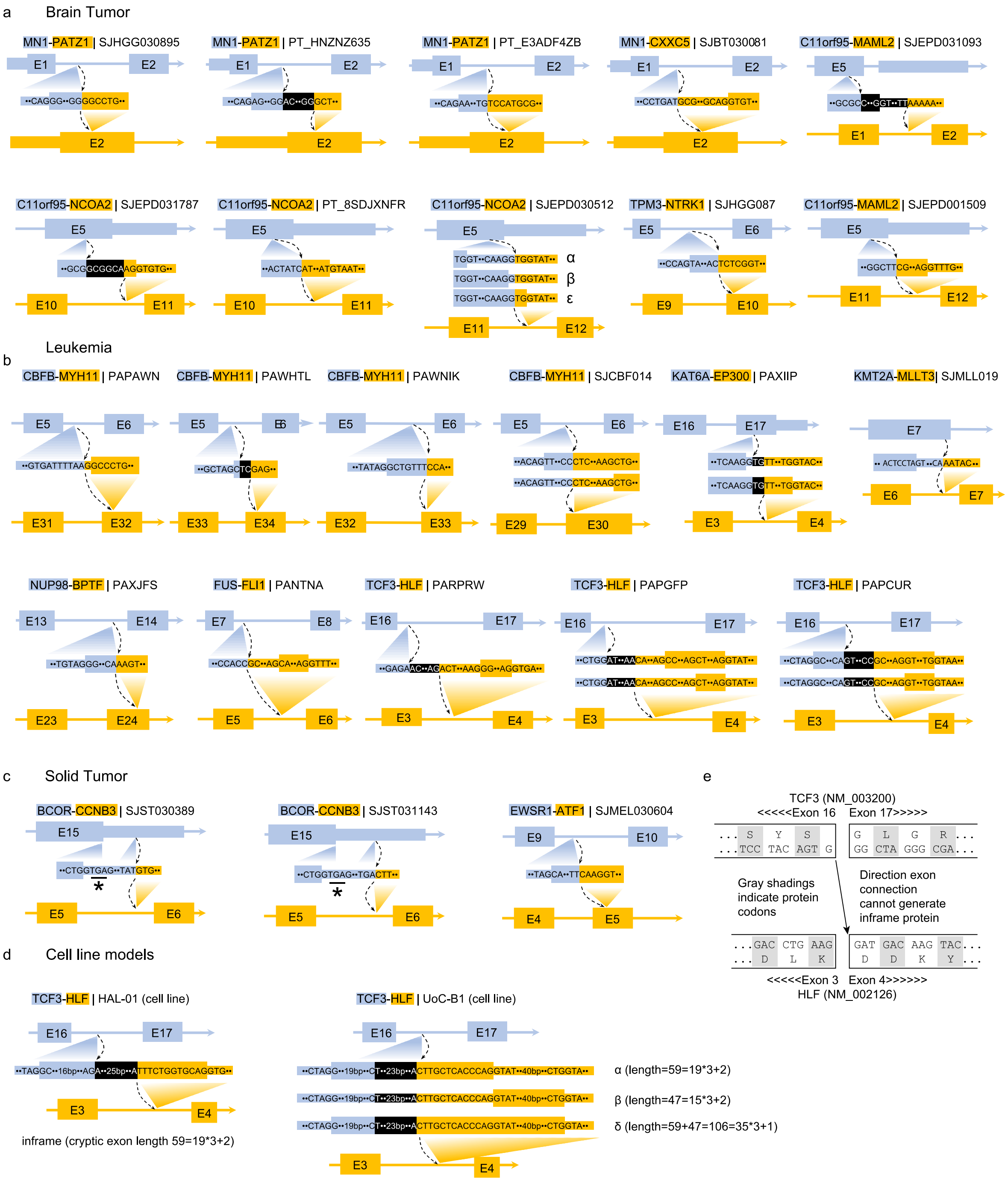
a
Brain Tumor
MN1-PATZ1 | SJHGG030895
MN1-PATZ1 | PT_HNZNZ635
MN1-PATZ1 | PT_E3ADF4ZB
MN1-CXXC5 | SJBT030081
C11orf95-MAML2 | SJEPD031093
C11orf95-NCOA2 | SJEPD031787
C11orf95-NCOA2 | PT_8SDJXNFR
C11orf95-NCOA2 | SJEPD030512
TPM3-NTRK1 | SJHGG087
C11orf95-MAML2 | SJEPD001509
α
β
ε
b
Leukemia
CBFB-MYH11 | PAPAWN
CBFB-MYH11 | PAWHTL
CBFB-MYH11 | PAWNIK
CBFB-MYH11 | SJCBF014
KAT6A-EP300 | PAXIIP
KMT2A-MLLT3| SJMLL019
NUP98-BPTF | PAXJFS
FUS-FLI1 | PANTNA
TCF3-HLF | PARPRW
TCF3-HLF | PAPGFP
TCF3-HLF | PAPCUR
c
Solid Tumor
BCOR-CCNB3 | SJST030389
BCOR-CCNB3 | SJST031143
EWSR1-ATF1 | SJMEL030604
d
Cell line models
TCF3-HLF | HAL-01 (cell line)
inframe (cryptic exon length 59=19*3+2)
TCF3-HLF | UoC-B1 (cell line)
α (length=59=19*3+2)
β (length=47=15*3+2)
δ (length=59+47=106=35*3+1)
e
TCF3 (NM_003200)
<<<<<Exon 16
Exon 17>>>>>
Gray shadings indicate protein codons
Direction exon connection cannot generate inframe protein
<<<<<Exon 3
Exon 4>>>>>
HLF (NM_002126)

**Supplementary Fig. 8 CRISPR targeting in HAL-01.** Shown are NGS results for guides $g_1$ (non-template insertion in cryptic exon; panel **a**), $g_2$ (neo splice donor; panel **d**), $g_3$ (neo splice acceptor; panel **e**), $g_4$ (upstream negative control; panel **b**), and $g_5$ (downstream negative control; panel **c**). For panel **a**, the rate of on-target editing (On-Target), the rate of lethal and non-lethal on-target editing are shown as a heatmap for three replicates from day 3 to day 19. For panels **b** and **c**, instead of using lethal/non-lethal, a tag of frameshift/in-frame was given to each indel according to its length because the target region is genuine intronic (thus a negative control). For panels **d** and **e**, the induced indels that happened to fall into coding region and lead to frameshift of *TCF3-HLF* are categorized into "Coding" group. Indels that directly disrupt the splice donor site are categorized into "Loss" group. For panels **d** and **e,** the induced indel may leave a residual GT/AG site. We calculated binding affinity of such residual splice sites by using a position specific weight matrix (PWM) approach (see **Methods**). Indels were grouped to bins according to their binding affinity scores (e.g., <2, 3-4, etc.). In e, most of the induced indels targeting neo acceptor fall into the coding region, so that only the "Loss" category has a sharp decrease of NGS read abundance from ~15% at day 3 to ~0% at day 19 post editing. On the other hand, ~15% of editing resulted in splice acceptor binding affinity to fall in bin of 5-6 at day 3, and these editing has resulted in a better fitness of host cells so that the NGS read abundance increased to >50% at day 19, a >3-fold increase. The binding affinity of the acceptors after these indels are predicted using position weight matrix (PWM) approach (see **Methods**). Replicate 2 of day 13 did not generate enough NGS reads for analysis and is indicated by black color. Due to rounding error, the row sums can be 100 or 99. Source data are provided in sheet Supplementary Fig.8 in Source Data file.

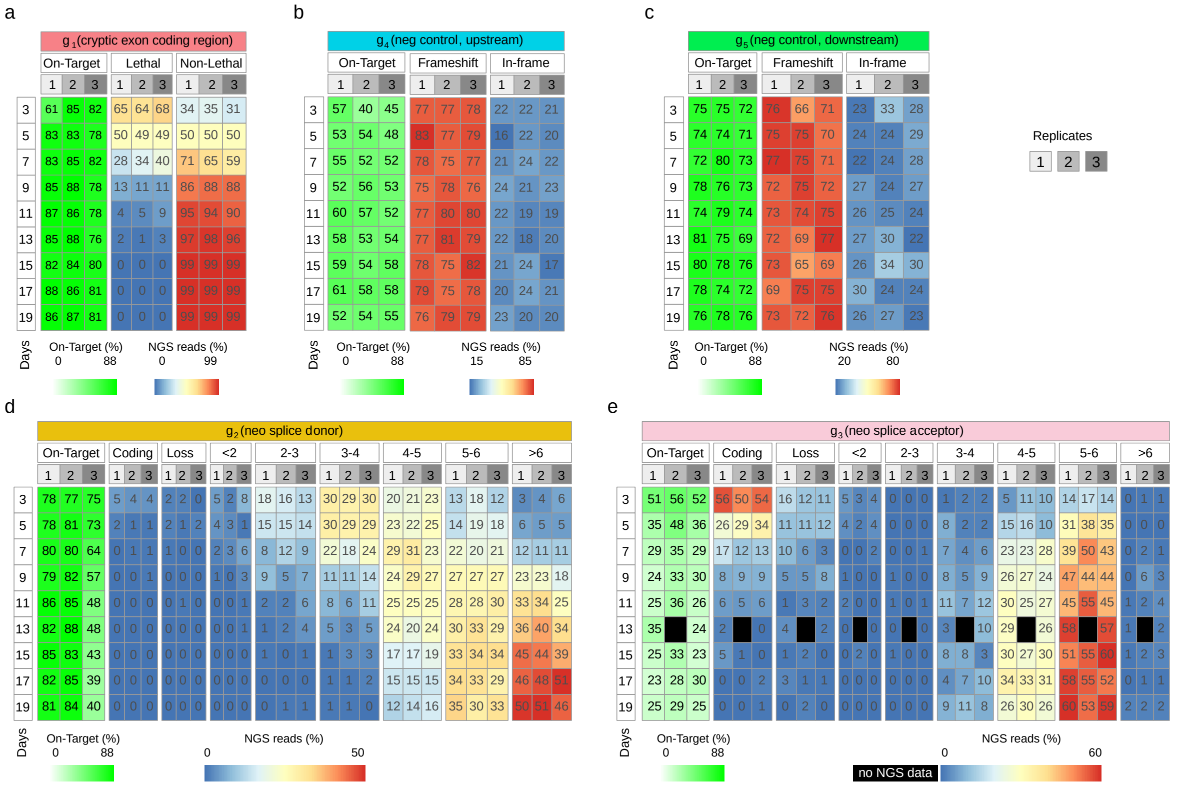

**Supplementary Fig. 9 CRISPR targeting in UoC-B1.** Shown are NGS results for designed guides $g_6$ (targeting isoform α; panel **a**), $g_7$ (targeting isoform β; panel b), double guides $g_6$+ $g_7$ (targeting isoforms α, β and δ; panel d). For each experiment, the rate of on-target editing (On-Target), the rate of lethal and non-lethal on-target editing are shown as a heatmap for three replicates from day 3 to day 19. For panel b, replicate 3 of day 11 did not generate sufficient NGS read and is indicated by black. In panel c, the putative effect of indel length combinations is analyzed for isoforms α, β, and δ, respectively, according to frame status (I=in-frame; O=out-of-frame). The final impact on host cells is indicated by lethality (Y=Yes, N=No). Indel length is presented as modulus of 3 (reminder of division by 3). Source data are provided in sheet Supplementary Fig.9 in Source Data file.

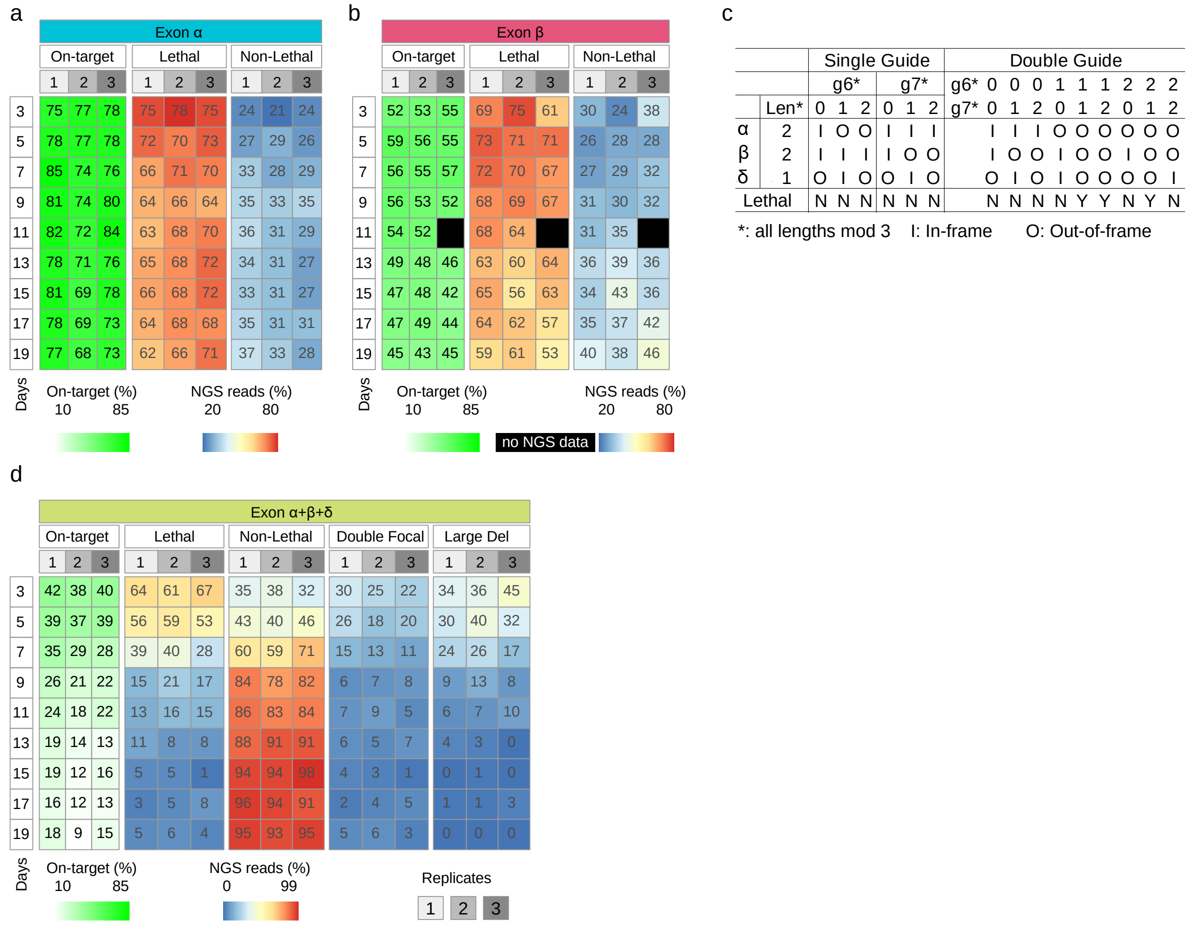

**Supplementary Fig. 10 Motif score distribution of true splice sites of human genes** (blue; from chromosome 19) versus non splice sites (gray, i.e., not defining exon/intron structure of human genes) for splice donor (top) and splice acceptor (bottom). Motifs are provided in Supplementary Data 20. Source data are provided in sheet Supplementary Fig.10 in Source Data file.

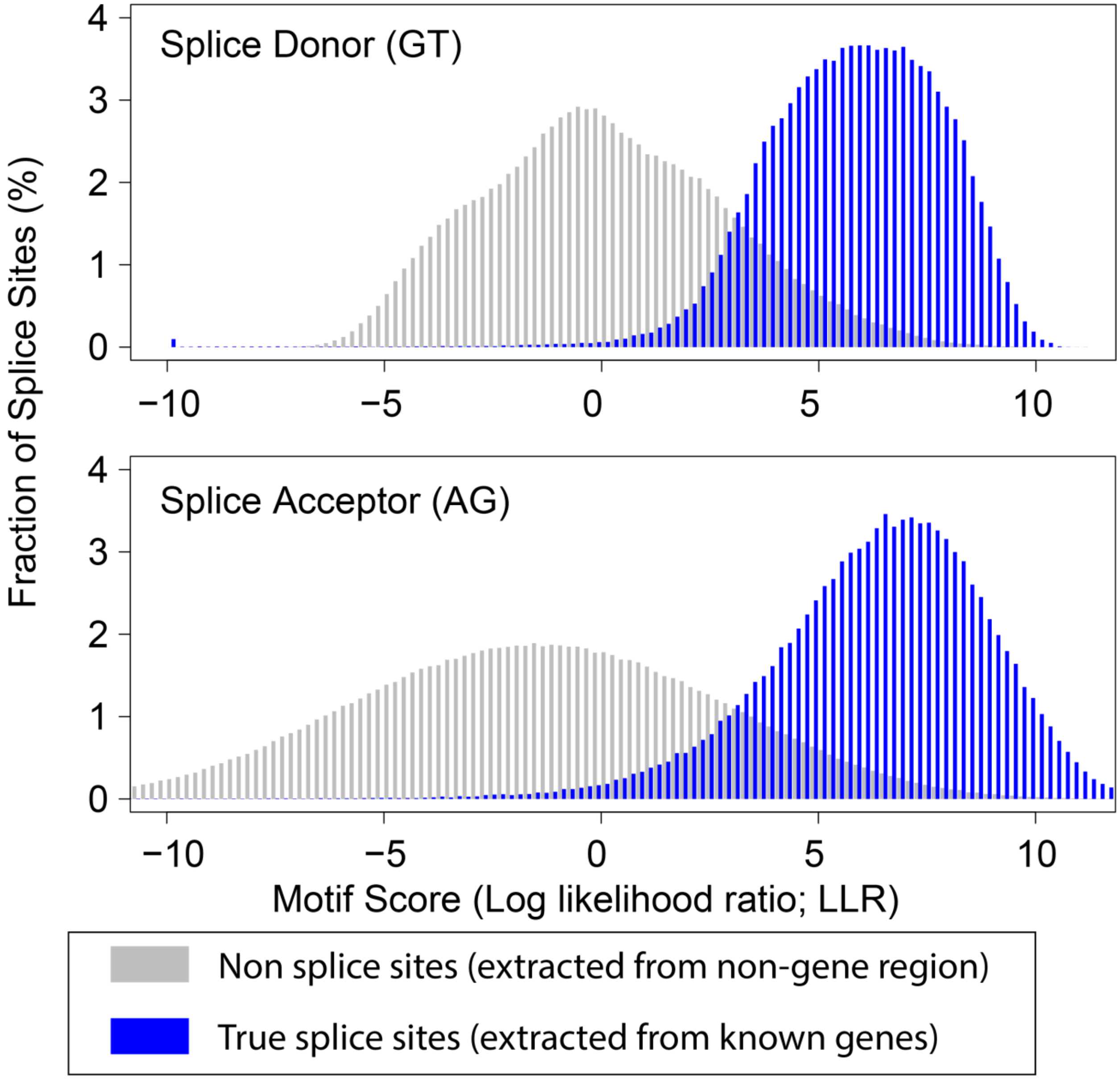

**Supplementary Fig. 11 Length distribution of induced indels.** Show are indels detected from guide RNAs $g_1$, $g_2$, $g_3$ in cell line HAL-01 (**a**), single guide RNAs $g_1$ and $g_2$ in cell line UoC-B1 (**b**) and double guide RNA $g_6$ + $g_7$ in cell line UoC-B1 (**c**). Over 95% of induced indels have length between -10 and 10 across all single guide targeting experiments, except the double guide in UoC-B1 that also generated large deletions of size around -55 bps (exon δ; panel **c**). Insertion appears to predominant our targeting over deletions and warrant further investigation. Only high confidence indel calls (with 3 or more supporting reads) are included in analysis. Source data are provided accordingly as sheet Supplementary Fig.11a and Supplementary Fig.11b-c in Source Data file.

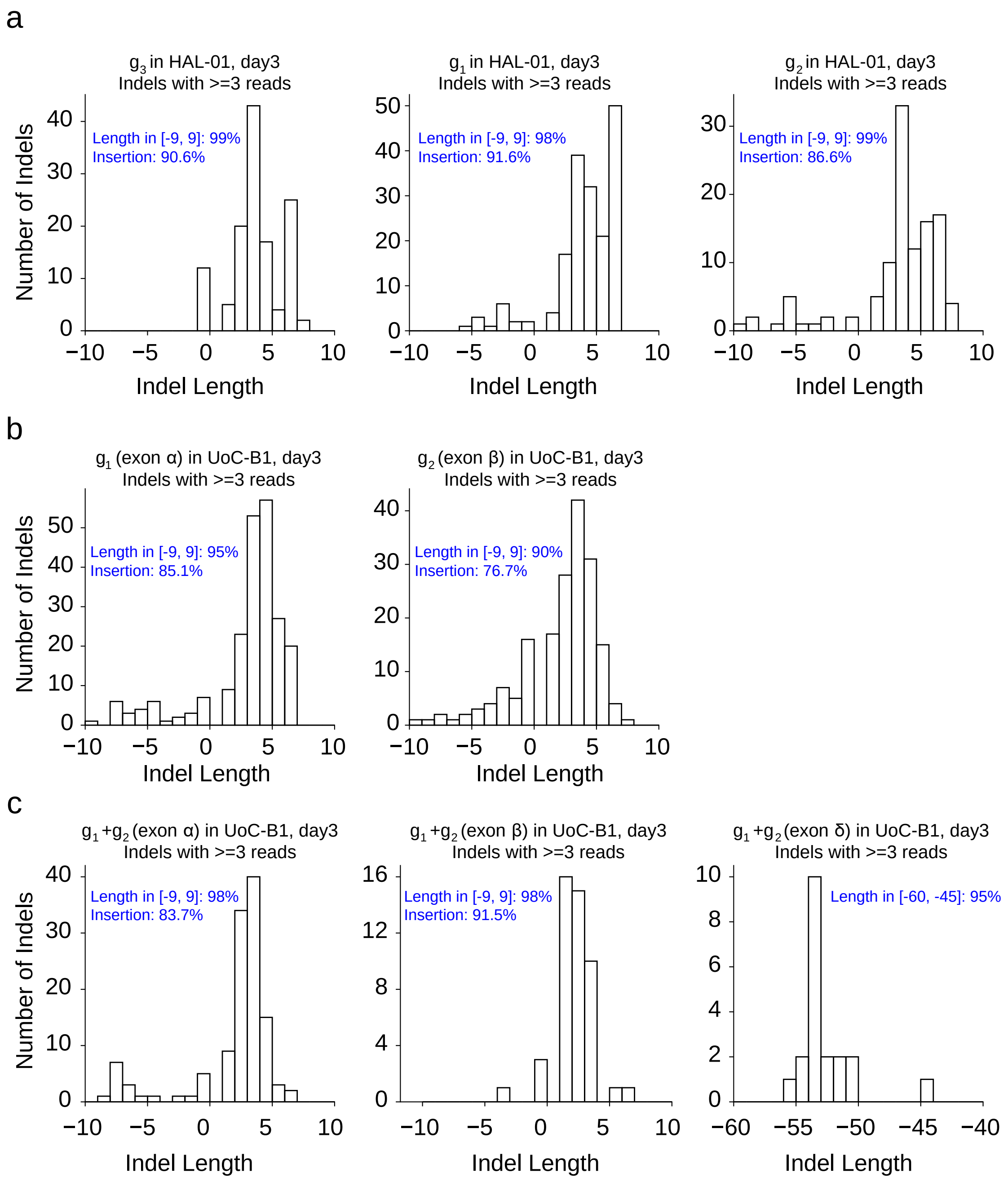

# Supplementary Notes (SN): Study design rationale

## 1. Clinically recognized oncogenic fusions that generate chimeric proteins

Since the discovery of *BCR-ABL1* fusion oncoprotein in Philadelphia-chromosome leukemia[1], classical cytogenetics method has revealed many additional cancer subtypes such as t(1;19)(q23;q13)[2] that was later determined to generate *TCF3-PBX1*[3], t(17;19)[4] that was later determined to generate *TCF3-HLF* [5] and t(11;22) (q24;q12) that was determined to generate *EWSR1-FLI1*[6]. With the advent of next generation sequencing technologies, oncogenic fusions are increasingly discovered in the past decade via simultaneously interrogating the genome (DNA sequencing) and the transcriptome (RNA sequencing) of tumors from patient cohorts of similar diagnosis, where DNA and RNA data cross validate each other. This include *C11orf95-RELA* (*C11orf95* recently renamed as *ZFTA*) in pediatric ependymomas (EPD)[7], *KIAA1549-BRAF* in pediatric low-grade glioma (LGG)[8]. Supporting evidence from both DNA (on the rearrangement) and RNA (on the generation of chimeric protein) is a critical feature of these findings.

## 2. Clinically recognized oncogenic fusions that leads to aberrant expression of proto-oncogenes

In addition to the above "conventional" oncogenic fusions where a chimeric fusion oncoprotein is generated, there is another category of "promoter-hijacking" fusions. In this category, a constitutively active promoter or enhancer region is brought to a proto-oncogene (via chromosomal rearrangement) that is typically silenced in corresponding lineage of the cancer cells. Such rearrangement leads to aberrant expression of the proto-oncogene. Prominent examples of this category include *CRLF2*/*DUX4*/*EPOR* aberrant expression via rearrangement to immunoglobulin heavy chain (*IGH*) region B-ALL[9,10], *TAL1*/*TAL2* aberrant expression via rearrangement to T-cell receptor region (*TCR*) in T-ALL[11], *GFI1* aberrant expression via intra-chromosomal rearrangements to active enhancers in medulloblastoma[12], *CRLF2* aberrant expression via intra-chromosomal rearrangement to *P2RY8* promoter[13], as well as our newly discovered *BCL11B* aberrant expression in lineage-ambiguous leukemia[14]. Because no chimeric proteins are generated, this fusion category is typically termed "promoter/enhancer-hijacking". Interestingly, other mutational mechanisms can also lead to such aberrant expression of proto-oncogenes. For example, the seminal work by Thomas Look and colleagues[15] has demonstrated that small insertions/deletions in enhancer regions of proto-oncogene *TAL1* can be sufficient to lead to its aberrant expression in pediatric T-ALL. Although corresponding tumors do not have chromosomal rearrangements (or fusion events) involving *TAL1*, we still consider these tumors as *TAL1* category.

## 3. Functional evidence of clinically recognized oncogenic fusions

Although experimentally challenging, putative oncogenic fusions such as *ZFTA-RELA* (also known as *C11orf95-RELA*) have recently been shown to be sufficient to drive pediatric ependymoma[16]. On the other hand, the success of imatinib on *BCR-ABL1*[17], and the genetic knockout of oncogenic fusions such as *TCF3-HLF* in this work, have demonstrated that these oncogenic fusions, or more precisely the fusion oncoproteins they encode, plays an essential role to the survival of host cancer cells, which forms the basis of the hypothesis "oncogene addiction" that posits on the therapeutic value of targeting these oncogenic fusions.

## 4. Clinically recognized oncogenic fusions being invariable to clonal evolution and initiating driver

Comparison of tumors collected at initial diagnosis and at relapse for pediatric leukemia[13,18] has reinforced the notion that subtype-defining oncogenic fusions are cancer initiating events[19]. In these studies, the oncogenic fusions are always conserved between diagnosis and relapse tumors, although other subclonal mutations (e.g., *CDKN2A* loss and *NT5C2* gain-of-function mutations) can be either eradicated or *de novo* acquired from diagnosis to relapse[13]. The clonal nature (i.e., being present in all cancer cells) of oncogenic fusions thus renders them ideal therapeutic targets.

## 5. Oncogene versus tumor suppressor gene (TSG)

In addition to the many oncogenic fusions mentioned above, extensive genome sequencing efforts in the past decade have led to the discovery of many additional significantly mutated genes also known as cancer drivers, in both adult[20,21] and childhood cancers[9,22]. In observation of these many cancer driver genes, Bert Vogelstein and colleagues[23] have pioneered the concept of classifying cancer driver genes into "tumor suppressor genes (TSG)" and "oncogenes", where a "TSG" is a gene that, when *inactivated* by mutation, increases the selective growth advantage of the cell in which it resides, while an "oncogene" is a gene that, when *activated* by mutation, increases the selective growth advantage of the cell in which it resides. Under this broad concept, the oncogenic fusions mentioned above belong to the category of "oncogene" because corresponding fusion oncoproteins are hyperactive. On the other hand, the well-known TSGs including *CDKN2A* and *RB1* [9,22] typically demonstrate inactivating (also known as loss-of-function) mutations, including gain of stop codon, protein-frame shifting, splice site altering, whole gene loss due to large deletion, or partial gene truncation due to focal deletion. A model of functional consequences on TSGs and oncogenes from diverse mutation types are illustrated in **Supplementary Fig. 12-17,** with data in figure adapted (Oct 15, 2022) from https://pecan.stjude.cloud/[24].

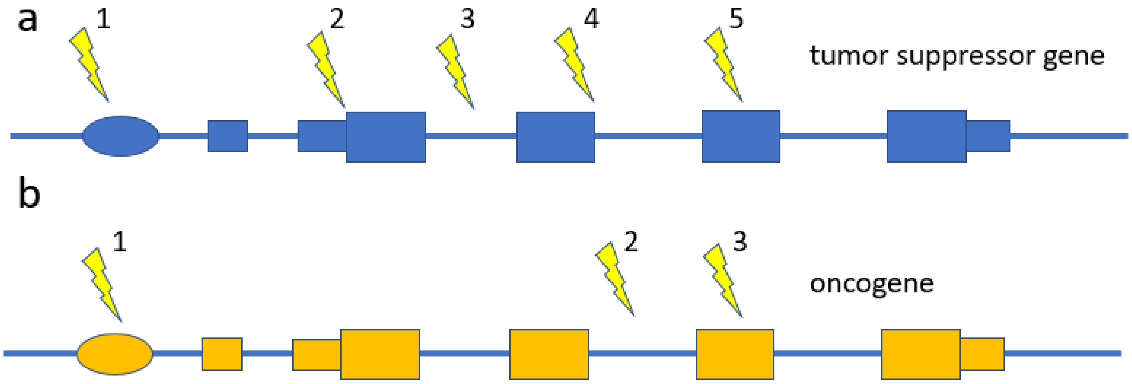


**Supplementary Fig. 12 Diverse mutation types to disrupt a tumor suppressor gene (TSG; a) and less diverse mutation types for hyperactivation (b).** In a TSG, a mutation (#1) that disrupts promoter or enhancer can lead to expression loss, a mutation (#2) that disrupts translation start codon ATG, a mutation (#3) that disrupt the gene structure via an intronic breakpoint, a mutation (#4) that disrupts the splice sites, and a mutation (#5) that disrupt the protein codon can all lead to loss of function (total gene deletion not illustrated). On the other hand, there are limited ways to make an oncogene hyperactive (panel **b**), which include a stronger promoter/enhancer via mutation #1, a stronger amino acid via mutation #3, or forming a chimeric protein via rearrangement mutation #2.

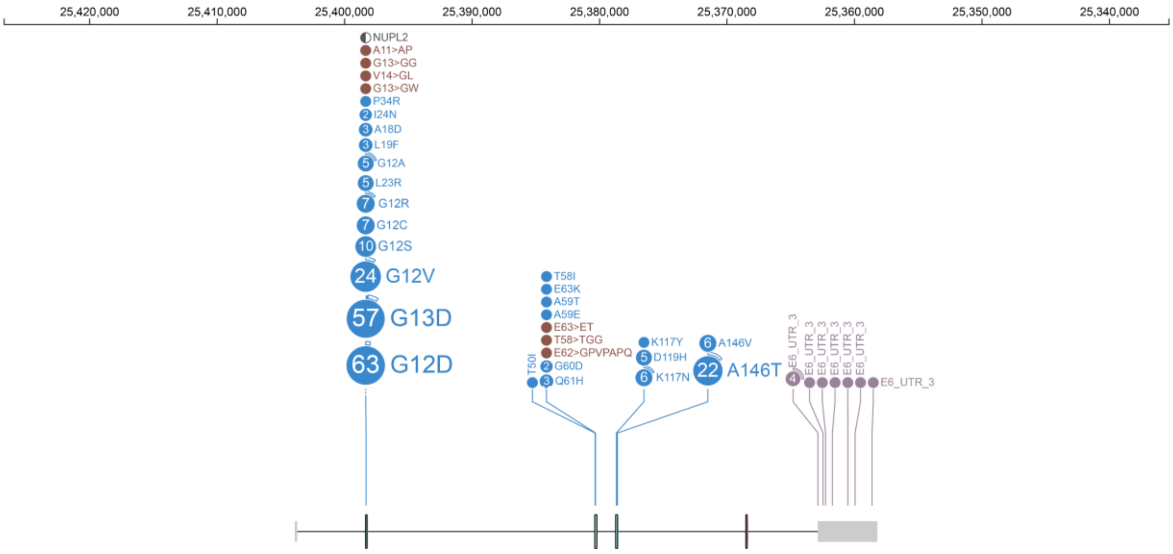


**Supplementary Fig. 13 Example oncogene *KRAS***. Chromosome coordinates (chr12) are shown on top and gene structure shown on bottom. *KRAS* mutations detected from pediatric cancers were shown as numbers that also indicate protein amino acid change. For example, there are 63 tumor specimens having mutations resulted in G12D, 22 tumor specimens having mutations resulted in A146T. Clearly, these protein coding mutations are in-frame and therefore can generate a mutant protein.

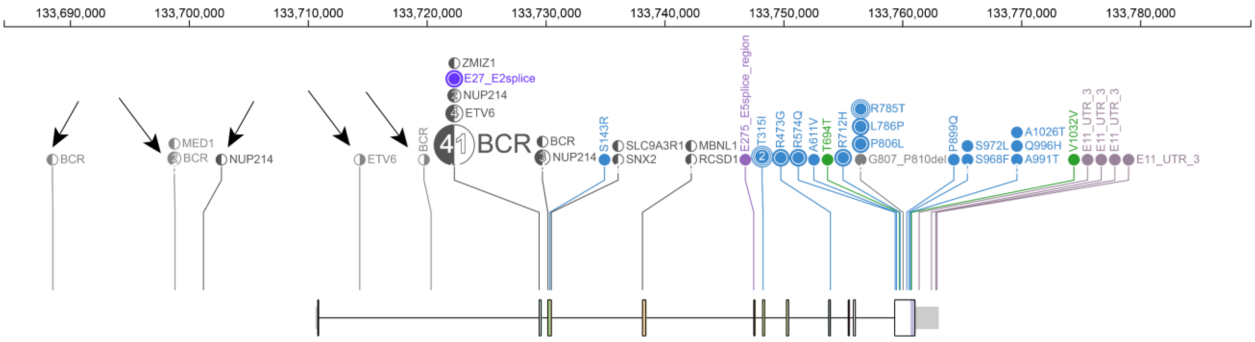


**Supplementary Fig. 14 Example oncogene *ABL1***. In addition to the few point mutations detected from thousands of pediatric cancers, a prominent observation in *ABL1* is fusions, including 41 *BCR-ABL1*, 4 *ETV6-ABL1*, and a few other rare fusions such as *RCSD1-ABL1*, all shown as half-white-half-black circles. Breakpoints aligned with exon boundaries represent RNA breakpoints (i.e., splice junctions) while breakpoints not aligned with exon boundaries represent DNA breakpoints (black arrows). While DNA breakpoints can sometime be detected from RNAseq data (in total RNAseq protocol where pre-mRNA are included, see **section SN 9** "**Predicting DNA breakpoints from RNAseq data**" in this **Supplementary Notes**), whole genome DNA sequencing typically ensure ascertainment of DNA breakpoints. On the other hand, DNA sequencing data typically cannot give definitive clue on RNA breakpoints (i.e., splice junctions) due to possibility of alternative splicing, such as *KMT2A* rearrangements in **Fig. 4d** of this work.

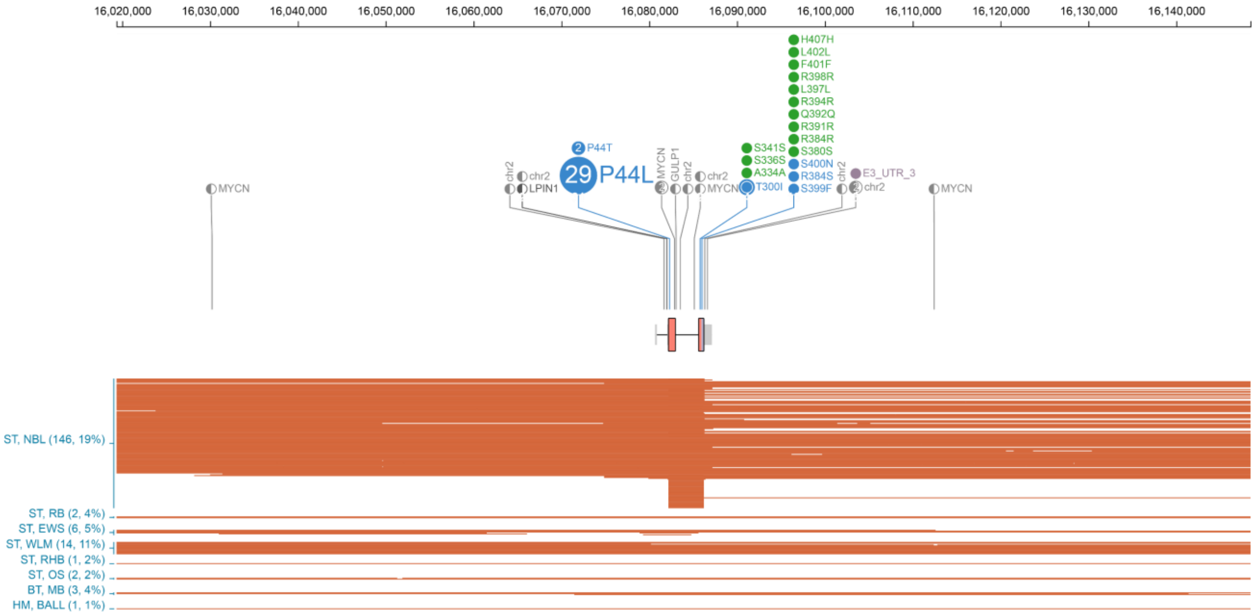


**Supplementary Fig. 15 Example oncogene *MYCN***. Beside P44L mutation in 29 tumor samples, *MYCN* does not have other highly recurrent alterations except copy number gain (red horizontal color bars in bottom) that are enriched in solid tumor (ST) neuroblastoma (NBL) that is detected in 19% of tumors (n=148). Further, *MYCN* amplification is also detected in Wilms tumor (ST, WLM) with frequency 11% (n=14). Note the common amplified region of *MYCN* and sometime the amplification can extend to far flanking regions of *MYCN*.

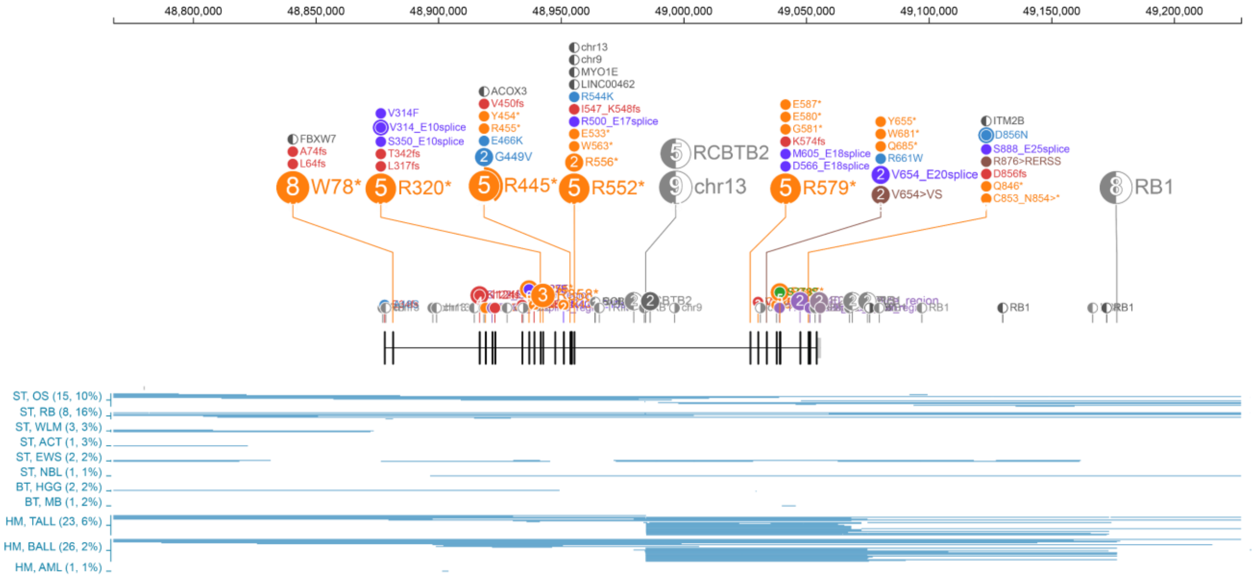


**Supplementary Fig. 16 Example tumor suppressor gene *RB1***. *RB1* gene has diverse mutation types in pediatric cancers, including stop gain mutations such as W78* in 8 specimens, R320* in 5 specimens. We also observed frameshifting mutations such as A74fs, L64fs, L317fs, D856fs. Moreover, the half-white-half-black circles indicate enrichment of structural rearrangements such as to gene *RCBTB2* in 5 patients

and another 9 patients to another region in chr13. Further, focal deletions were detected in pediatric T-ALL (6%) and B-ALL (2%) that removed last several exons of *RB1*.

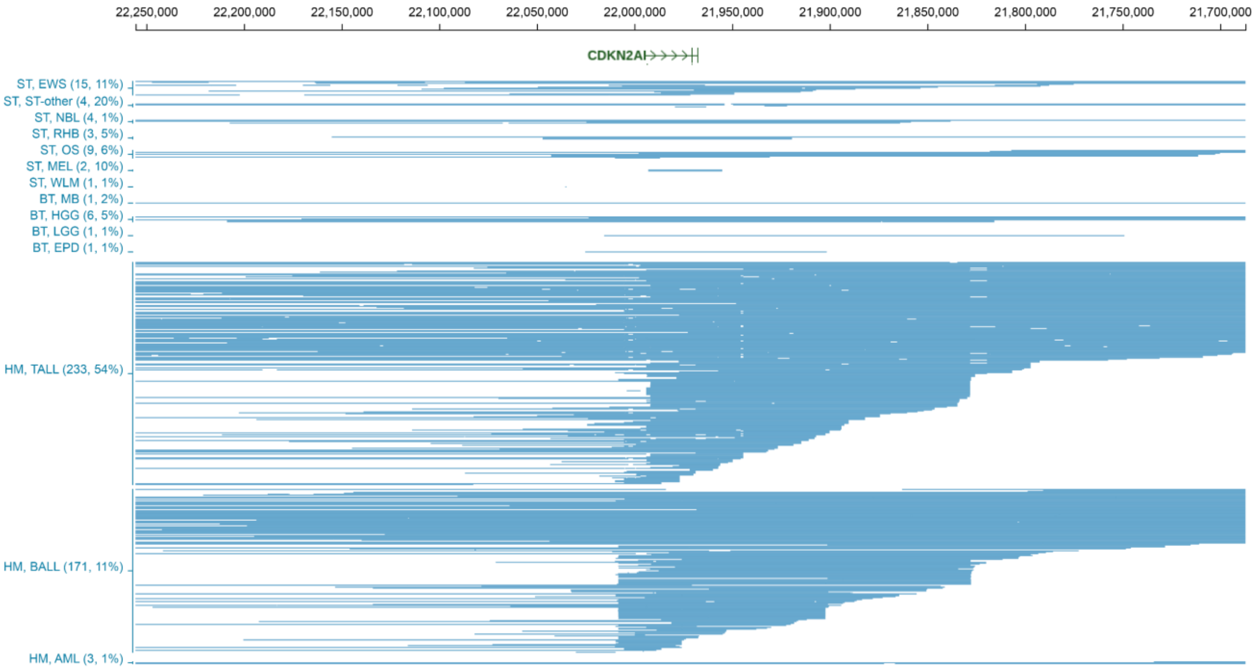


**Supplementary Fig. 17 Example tumor suppressor gene *CDKN2A***. Unlike TSG *RB1*, *CDKN2A* is enriched with copy number loss in pediatric T-ALL (54%) and B-ALL (11%). Although in some tumors the detection can be so focal that only few exons are affected (in this case it is possible to detect a truncating "fusion" from RNAseq data), in many tumors the size of deletion can be as big as arm level so that no truncating "fusion" transcripts are expected in RNAseq data.

## 6. Clinically recognized fusion-negative samples

Although oncogenic fusions have been routinely used for clinical subtyping, not all human cancers are fusion positive. For example, in pediatric B-ALL it has long been known that fusion-negative subtypes exist, including hyperdiploid (that with >50 chromosomes) and hypodiploid (those with <45 chromosomes) B-ALL[25]. In pediatric neuroblastoma, extensive efforts in the study of whole genome, exome, and transcriptome sequencing data have not identified clinically meaningful oncogenic fusions for most samples, except the well-known high *MYCN* amplification in ~20% of patients[9,26,27]. In malignant rhabdoid tumours, *SMARCB1* homozygous loss is the only hallmark of nearly all patient tumors[28]. Similarly, *RB1* homozygous loss is the only hallmark of nearly all retinoblastoma tumors[29]. Clearly, candidate fusions detected in tumors of fusion-negative subtypes such as hyperdiploid B-ALL, neuroblastoma, rhabdoid or retinoblastoma tumors are more likely passenger events, if not artefacts, and scrutiny is warranted before accepting them as a true oncogenic fusion, as will discussed in section **SN 11g** on mutual exclusivity pattern among oncogenic fusions. This data highlights the critical need of knowledge on well-defined tumor subtypes to ensure scientific rigor in reporting novel oncogenic fusions. In fact, clinically-relevant novel fusion-negative subtypes continue to be discovered, such as the novel subtype of *UBTF*-ITD in pediatric AML among the known clinical fusion-negative subtypes of *NPM1* and *CEBPA*[30].

## 7. Remarks on clinically recognized oncogenic fusions

The above data highlights a few characteristics of clinically recognized oncogenic fusions such as *BCR-ABL1*: 1) to date all of these fusions are in-frame and activating (i.e., TSG does not belong to the category of oncogenic fusions); 2) promoter/enhancer-hijacking can be regarded as a different category of oncogenic fusion because they do not generate chimeric proteins; 3) these fusions are subtype-defining so that typically we see no more than one fusion per tumor, also known as mutual exclusivity rule[30] that will be discussed in section **SN 11g**; 4) despite extensive clonal evolution during the course of the life span of a tumor, subtype-defining oncogenic fusions typically remain intact; 5) like *ZFTA-RELA* (also known as *C11orf95-RELA*) and *TCF3-HLF*, these fusions are expected to be functionally sufficient and necessary to the host cancer cells; 6) not all human cancers are expected to have oncogenic fusions. Interestingly, to date all clinically recognized oncogenic fusions in pediatric cancers have supporting evidence from both DNA and RNA sequencing data whenever both data types are available, highlighting a critical bioinformatic pattern during technical evaluation of candidate oncogenic fusions.

## 8. Study design of this work

The above molecular mechanistic insights lead us to following strategy in this study design.

**8.a) Tumor suppressor genes**. Due to the diverse mutation types (including substitutions (SNVs), small insertion/deletions (Indels), copy number loss (CNVs), or structural alterations (SVs)) that can all lead to loss-of-function, we always rely on DNA sequencing (especially whole genome sequencing) to definitively ascertain the mutation status for TSGs. Although occasionally truncating mutations can be detected in RNAseq, we deem a whole-genome sequencing cohort would better serve the goal of comprehensively and unbiasedly studying etiology. In fact, we are currently drafting a manuscript on the signatures of rearrangements (SVs) using whole genome sequencing (WGS) in >1,500 pediatric cancer patients. With this consideration, we decided to NOT include tumor suppressor gene in this study, which is designed to focused on oncogenic fusions like *BCR-ABL1*. However, in response to Reviewer #2's request, we analyzed highly frequent *CDKN2A* and *NBAS* truncating fusions in section **SN 12**.

**8.b) Oncogenic fusions in the category of promoter/enhancer hijacking**. In this category, a rearrangement can bring a strong promoter/enhancer to an otherwise silenced proto-oncogene and lead to its aberrant expression. When the novel promoter is far, the proto-oncogene may start its transcription from its own transcription start site, thereby leaving no split reads or discordant read pairs in RNAseq data for bioinformatic detection (**Supplementary Fig. 18a**). On the other hand, the transcripts may contain part of the novel promoter sequences when the novel promoter is closer (**Supplementary Fig. 18b**). Moreover, it is also possible that a point mutation in the native promoter can convert it to a strong active promoter (such as *TAL1* in pediatric T-ALL[15]) and lead to aberrant expression (**Supplementary Fig. 18c**)— biologically this scenario is not promoter/enhancing *per se*. Clearly, without DNA (preferentially whole genome) sequencing data, scenarios a) and c) cannot be resolved by transcriptome sequencing and, a forced analysis will result in biased conclusions that does not meet our scientific rigor. Instead, such patterns are best studied in our ongoing project on the signatures of rearrangements (SVs) using whole genome sequencing in >1,500 pediatric cancer patients. Nevertheless, we provided results on known oncogenic fusions (*CRLF2*, *DUX4*, *EPOR*, *BCL11B,* **Supplementary Data 26-30**) in promoter/enhancer-hijacking category to address Reviewer #2's request though we did not perform systematic discovery.

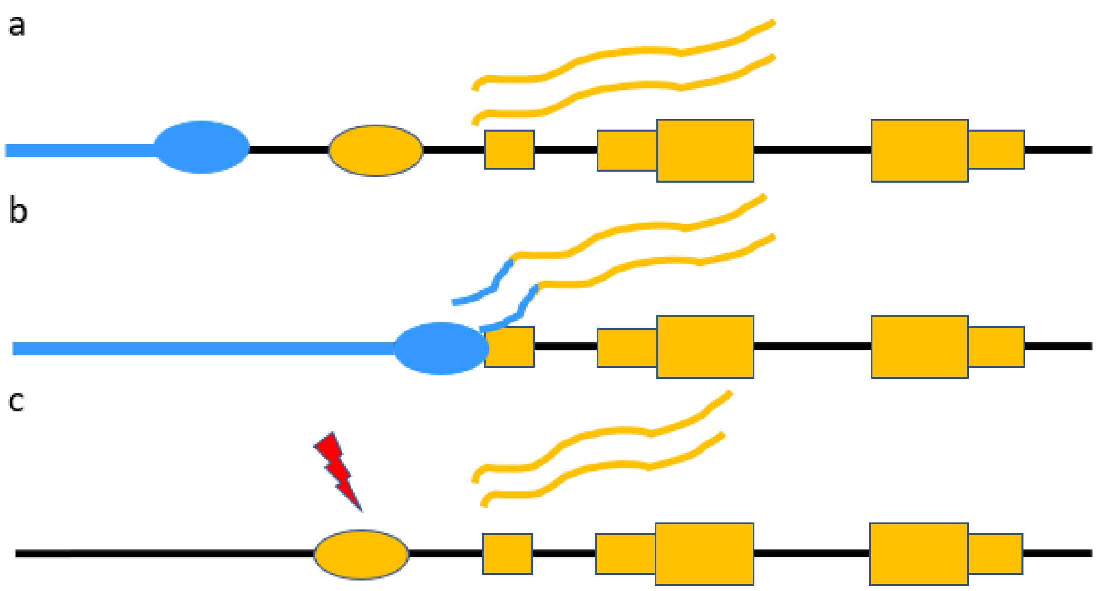


**Supplementary Fig. 18 Promoter/enhancer-hijacking.** In hypothetical scenario (a), the blue chromosome (and a strong enhancer/promoter highlighted by blue oval) was brough proximity to the orange proto-oncogene and lead to its aberrant expression. Transcription only involves the proto-oncogene due to the space between the blue promoter and orange gene. In (b), the blue promoter contacts the transcription start site of orange gene, so that the transcription involves both the proto-oncogene and a small part of the blue chromosome. In (c), a point mutation in the promoter region may convert it to a strong active promoter to initiate the proto-oncogene without a fusion event (such as *TAL1* enhancer mutation in Mansour et al (2014)[15]. Split reads or discordant read pairs are expected for scenario (b) but not scenario (a) or (c). DNA (preferentially whole genome) sequencing are needed to ascertain the fusion status. Nevertheless, the aberrant high expression of such proto-oncogene typically can help ascertain the tumor subtype.

**8.c) Conventional oncogenic fusions that generate chimeric proteins**. As shown in our **Fig. 1a**, oncogenic fusions that generate chimeric proteins are obligated to have split read or discordant read pair signals in RNAseq data, either polyT protocol or total RNA protocol. It is this exact category that our large cohort of 5,190 RNAseq datasets can be used to generate scientifically rigor discoveries.

## 9. Predicting DNA breakpoints from RNAseq data

In this work, in addition to RNA junctions, we attempted to detect DNA breakpoints (here termed d-event) from RNAseq data to interrogate the uniformity of DNA breakpoints in relative intronic regions. As shown in the model of **Supplementary Fig. 19**, although RNA splicing breakpoints (here termed m-event; also known as "fusion" or splice-junction) are guaranteed to be observed in mRNA species that have underwent splicing, theoretically d-events are only observed in total RNA sequencing but NOT in poly(T)-based mRNA sequencing. As a sanity check, we compared our d-event detections in RNAseq data against the ground truth d-events defined in DNA (whole genome) sequencing datasets and demonstrated that 91% of our detections are within 5-bp of ground truth (**Supplementary Fig. 3a**). The accuracy revealed by this sanity check enables us to reach reliable conclusion that DNA breakpoints are uniformly distributed in relative introns of oncogenic fusions.

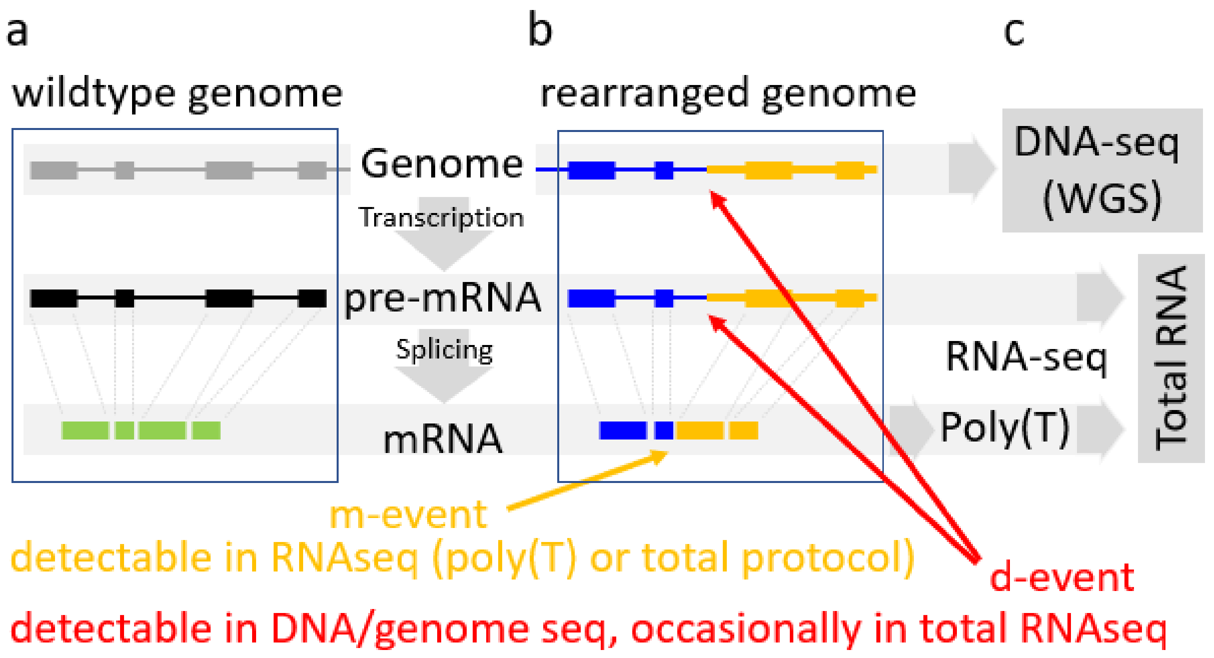


**Supplementary Fig. 19 Detecting DNA and RNA breakpoints from next generation sequencing data.** (a) During gene expression, genetic information encoded in wildtype human chromosomes (gray, thin line indicates intron/intergenic region, thick boxes indicate exons) are first transcribed into pre-spliced transcripts (pre-mRNA; black), which is in turn spliced (to remove introns and retain exons) to generate mature RNA species (mRNA, green). (b) This Central Dogma also applies to cancer genome (here blue and orange indicate two different chromosomal regions joined together by rearrangement). In this model, an intronic rearrangement (here termed as "d-event" to stress it is observed from DNA) happened between the two involved genes shown in blue and orange in the cancer genome. This d-event is observable in pre-mRNA but typically is not observed in mRNA because the intronic regions are spliced out. On the other hand, in mRNA, the rearrangement is manifested as splicing junctions (here termed "m-event" to indicate it is observed in mRNA; commonly referred to as "fusion" events) which typically are not directly observed in the DNA of the cancer genome although biological inference is possible (with alternative splicing being the confounding factor in consideration). (c) In next generation sequencing, we can perform DNA sequencing (such as whole genome sequencing (WGS), targeted capture) to detect d-events and RNA sequencing to detect m-events. Earlier RNA sequencing practices typically utilize poly(T) protocol, which can only interrogate mRNA species and therefore can NOT be used to detect d-events. Recent RNA sequencing practices typically utilize total-RNA protocol, which can simultaneously interrogate mRNA species and pre-mRNA species and enables simultaneous detection of d-event (if the total RNA contains sufficient pre-mRNA species and therefore is not guaranteed) and m-event.

## 10. Mutual exclusivity of subtype-defining genetic alterations such as oncogenic fusions

It has long been recognized that pediatric cancers can be subtyped using genetic alterations. In childhood B-ALL, clinically well-defined subtypes[25] include hyperdiploid and hypodiploid caused by chromosomal gains and losses, respectively; oncogenic fusions/translocations including *BCR-ABL1*, *KMT2A* rearrangements, *ETV6-RUNX1*, etc. This data indicates two critical facts: 1) a childhood tumor may harbor no oncogenic fusions, because genetic alterations such as hyperdiploid and hypodiploid can

also define subtypes. This is also true in other childhood cancers. For example, in neuroblastoma high *MYCN* amplification defines a genetic category. 2) a functional oncogenic fusion is sufficient to drive a distinct transcriptional program so that corresponding subtypes are well separated from other subtype, as clearly shown in childhood B-ALL[31], AML[30]. These two facts further imply mutual exclusivity among oncogenic fusions: typically, no childhood tumor can harbor ≥2 functional oncogenic fusions. To illustrate mutual exclusivity, we first analyzed 63 clinically well-defined oncogenic fusions (termed "**training set**" hereafter) including 1) leukemias: *BCR-ABL1*, *CBFA2T3-GLIS2*, *CBFB-MYH11*, *DEK-NUP214*, *EBF1-PDGFRB*, *EP300-ZNF384*, *ETV6-ABL1*, *ETV6-RUNX1*, *FUS-ERG*, *HNRNPH1-ERG*, *KAT6A-CREBBP*, *KAT6A-NCOA2*, *KMT2A-AFDN*, *KMT2A-AFF1*, *KMT2A-ELL*, *KMT2A-MLLT1*, *KMT2A-MLLT10*, *KMT2A-MLLT11*, *KMT2A-MLLT3*, *KMT2A-MYO1F*, *KMT2A-SEPTIN6*, *KMT2A-USP2*, *MEF2D-BCL9*, *DDX3X-MLLT10*, *NIPBL-HOXB9*, *NPM1-MLF1*, *NUP214-ABL1*, *NUP98-KDM5A*, *NUP98-NSD1*, *PAX5-AUTS2*, *PAX5-C20orf112*, *PAX5-CBFA2T3*, *PAX5-JAK2*, *PICALM-MLLT10*, *RBM15-MRTFA*, *RUNX1-RUNX1T1*, *SMARCA2-ZNF362*, *SSBP2-CSF1R*, *TCF3-HLF*, *TCF3-PBX1*, *TCF3-ZNF384*, TEC-*MLLT10*, *MYB-GATA1*; 2) brain and solid tumors: *KIAA1549-BRAF*, *C11orf95-RELA*, *FGFR1-TACC1*, *FGFR3-TACC3*, *TPM3-NTRK1*, *YAP1-FAM118B*, *YAP1-MAML2*, *YAP1-MAMLD1*, *CLIP1-ROS1*, *EWSR1-ERG*, *EWSR1-FEV*, *EWSR1-FLI1*, *EWSR1-WT1*, *ETV6-NTRK3*, *MYB-QKI*, *PAX3-FOXO1*, *PAX3-NCOA1*, *PAX7-FOXO1*, *SPTBN1-ALK*, *PPP1CB-ALK*. A patient with these oncogenic fusions detected by ≥2 of the 4 methods is called positive for corresponding oncogenic fusion. As it turned out, we detected these oncogenic fusions in 1,743 out of 5,190 patients, and only 4 patients (0.23%) have ≥2 oncogenic fusions (**Supplementary Data 29**). This data clearly demonstrates the exclusivity pattern among subtype-defining oncogenic fusions.

## 11. Integration of predictions from 4 fusion calling methods

The four selected fusion calling methods (Arriba, Cicero, FusionCatcher, and STAR-Fusion) each produced 5,781,630 (230,565 for STAR-Fusion, 252,718 for Arriba, 1,632,086 for Cicero and 3,666,261 for FusionCatcher) of predictions that are challenging to manually review. For this, we developed a majority voting strategy to enable efficient and reproducible analysis.

### 11.a) Ambiguity in chromosomal coordinate difference between calling methods

We first studied potential ambiguity in the chromosomal coordinates between calling methods. For this, we defined two fusion candidates (of the same sample) to be the same event if involving breakpoints are within $K$ base pairs. We varied $K$ from 100 to 50, 20, 10, 5, 2, and 1. As can be seen from **Supplementary Fig. 20a**, the number of shared fusion predictions remained robust till $K$=5 for all pairwise comparisons. Therefore, we choose to use $K$=10 to determine whether two fusion candidates are same events. In this way, we classified all >5.7 million predicted candidate fusions from these methods into 1) 6,431 with 4 votes; 2) 9,917 with 3 votes; 3) 65,013 with 2 votes; and 4) 5,443,485 with 1 vote (**Supplementary Fig. 20b**).

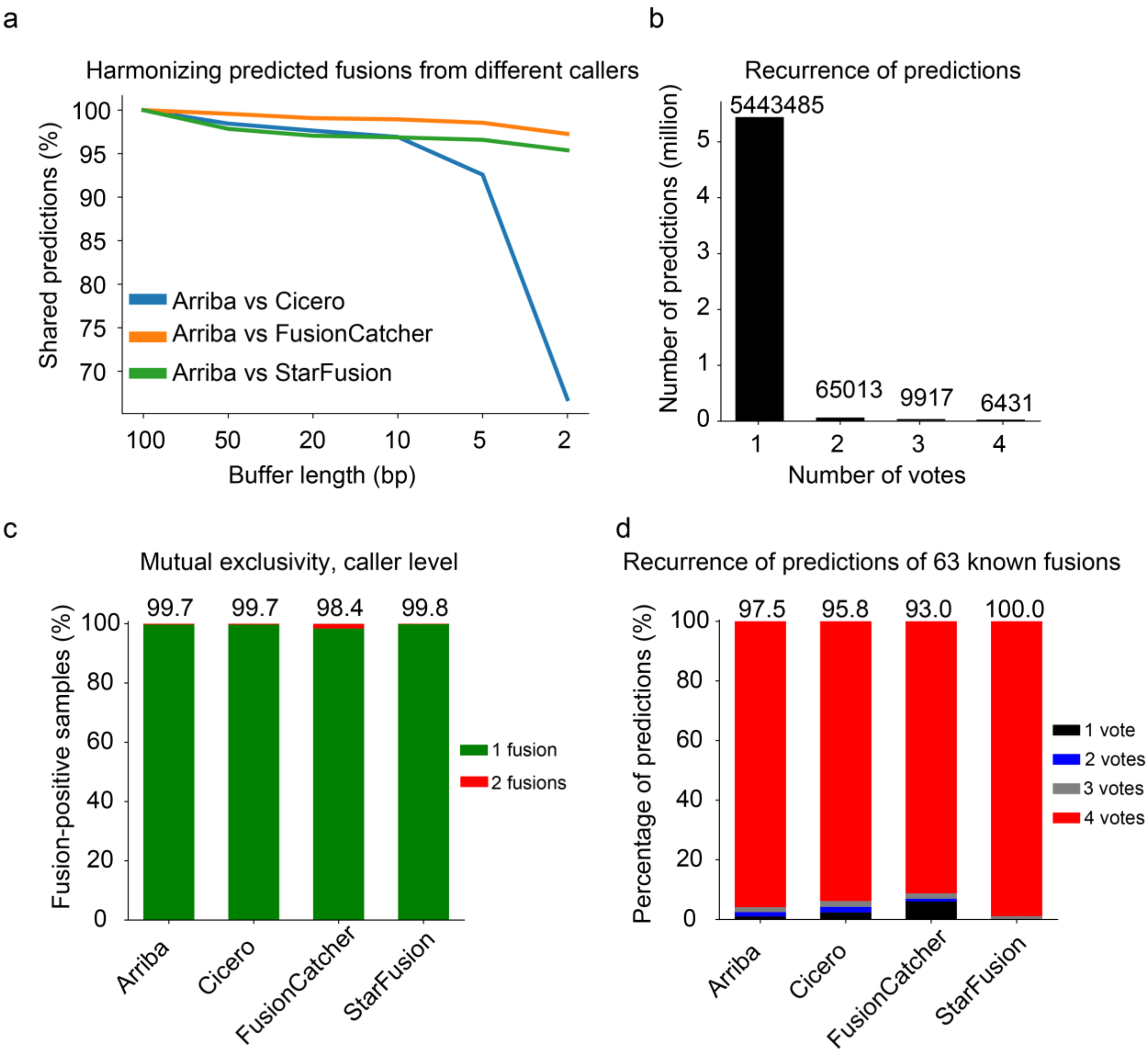


**Supplementary Fig. 20 Identifying oncogenic fusions from 4 prediction methods (Arriba, Cicero, FusionCatcher, and STAR-Fusion) by majority voting**. (a) Harmonization of fusion predictions. For chromosomal coordinates of same fusion events frequently differ between methods. We defined two predictions to be same events if the difference is within a predefined buffer length (from 100bp to 2bp) and the data suggests 10 to be a good cutoff. Because FusionCatcher and Cicero each have more than 1 million predictions, and because we will use majority voting, we choose to compare Arriba against other methods to determine the cutoff. (b) Recurrence of predictions. With the cutoff 10bp, we tallied frequency of predictions with 1, 2, 3, or 4 votes from different methods. Consistent with expectation, only 6,431, and 9,917 predictions have 4, 3 votes, respectively. This number (9917+6431=16348) of predictions are amenable for manual review. Therefore, we next tried to establish the enrichment of clinically relevant oncogenic fusions in these two bins by using 63 clinically well-known oncogenic fusions (c, d). First, we found that essentially all (>99.7%) fusion-positive tumors have exactly 1 oncogenic fusion (c), which is known as "mutual exclusivity rule". Second, we found that >93% of oncogenic fusions have 3+ votes. This data clearly established the enrichment of clinical-relevant oncogenic fusions within the high-vote bins, thus allowing us to efficiently analyze the whole cohort with

a low false negative rate (<7%). Source data are provided accordingly as sheet Supplementary Fig.20a, Supplementary Fig.20b, Supplementary Fig.20c and Supplementary Fig.20d in Source Data file.

### 11.b) Primary and secondary calling of the same fusion in a sample and their votes

As illustrated in **Fig. 4**, oncogenic fusions can be subjected to alternative splicing, which in turn results in multiple fusion candidates for fusion detection methods. Clearly, transcript isoforms with low read supports are less likely to be detected by multiple methods. To account for such multi-calling, we classified fusion isoforms detected from the same sample into two categories: 1) "Primary call" has the highest read support; 2) all other calls of the same fusion are termed "Secondary calls". We then studied the enrichment of clinically defined well-known fusions (the **training set** in section **SN 10**) within the voting categories defined in section **SN 11.a**, for Primary and Secondary calls. Using Arriba as an example (**Supplementary Fig.20c-d**), 1% and 1.5% of oncogenic fusion events has only 1 and 2 vote, respectively. As a result, 97.5% of oncogenic fusion events have 3 or 4 votes, indicating the significant enrichment of information in high vote bins for Arriba. Taken together, in overall 4 fusion detection methods, >93% of primary calls (that of FusionCatcher; >95.8% for all other three methods) are in the 3-vote and 4-vote categories across all four methods. Therefore, we decided to focus on the categories of 3-vote and 4-vote for discovery of novel oncogenic fusions, which can result in <7% false negatives. To further reduce impact of these potential 7% false negatives, we will check the newly discovered fusions in the 1-vote and 2-vote categories as detailed in section **SN 11.e**. This iterative process can be applied multiple times when effort permits.

### 11.c) Establishing "blacklists" using exclusivity pattern

As illustrated in section **SN 11.b**, 1,894 samples are determined to harbor a well-known oncogenic fusion. By using the mutual exclusivity rule among oncogenic fusions, we collected all fusion candidates (except the known oncogenic fusions) from these samples to establish "negative control", which is frequently referred to as "blacklist" in the field of cancer genomics, such as that used by Arriba[32].

### 11.d) Manual review of candidate fusions

The blacklist approach in section **SN 11.c** resulted in 7,427 (**Supplementary Data 21**) and 296 (**Supplementary Data 22**) fusion candidates with 4 and 3 votes, respectively. Manually review resulted in 218 additional oncogenic fusions (affecting 275 patients; **Supplementary Data 23**) involving well-known fusion genes including *BRAF*, *FGFR1/3*, *RAF1*, *ROS1* etc. Literature support of these rare oncogenic fusions are provided in **Supplementary Data 23**. There are 307 remaining in-frame fusions (affecting 218 patients; **Supplementary Data 24**) for which additional recurrence or functional evidence (i.e., lack of supporting literature) are needed to accept them as oncogenic fusions---these fusion candidates are labeled as "Tier2 Detection" and are not included in our formal analysis. A few notable criteria for our manual review are listed below (also detailed in captions of **Supplementary Data 21-22**): i) the candidate fusion involved well-recognized tumor suppressor genes (i.e., loss-of-function alterations, see section **SN 8a**) such as *NF1*, *PTEN*, *RB1*, *CDKN2A*, etc. are not considered oncogenic fusions. ii) well-known promoter-hijacking fusions (e.g., *CRLF2*, *DUX4*, *EPOR* etc.) are reviewed separately in **Supplementary Data 26-30**. iii) low quality predictions as indicated by fusion detection methods (such as "low" and "medium" confidence and "readthrough" by Arriba), low mutant read count or low allele fraction, ambiguous mapping, or a frameshift version of the oncogenic fusion that is labeled as "byproduct". Interestingly, complex rearrangement[33] (also known as 3-way fusions) between *KMT2A*,

*MLLT10* and *PIP4K2A* was detect in a sample (SJAML065570_D1, **Supplementary Data 21**) and we only considered *KMT2A-MLLT10* in this patient. Although this procedure started with 63 clinically well-known oncogenic fusions, the later steps are *de novo* discovery methods and ensures unbiased detection of oncogenic fusions.

### 11.e) Systematic identification of oncogenic fusions in all samples

With the comprehensive list of oncogenic fusions, including the **training set** (section **SN 10**) and 218 newly identified rare oncogenic fusion gene pairs (**SN 11d**), we extracted all predictions with 2 or more votes to maximize our detection. Upon manual review (**Supplementary Data 31**), we determined 2239 samples (involving 2005 patients) to be positive for chimeric oncogenic fusions.

A similar process was applied for promoter-hijacking oncogenic fusions involving *CRLF2*, *DUX4*, *EPOR*, *BCL11B*, *MECOM*, *HOXA*/*HOXB*, excluding *MYC* (**Supplementary Data 26-30**). With this category, we determined 254 samples (involving 240 patients) to be positive for oncogenic fusions (**Supplementary Data 30)**.

### 11.f) Determining orientation of oncogenic fusions

Due to frequent balanced translocations (at DNA level), in RNAseq we can frequently detect reciprocal fusions for the same oncogenic fusions, such as *RUNX1-ETV6* in *ETV6-RUNX1* patients. To account for this possibility, we counted the number of patients/samples supporting the two possible orientations. We discovered that the larger of these two numbers matches the known oncogenic fusion orientation in literature for all 52 fusion gene pairs detected in ≥4 patients/samples. For oncogenic fusions with recurrence <3, 143 fusion gene pairs are discovered with one 1 orientation. Orientation of the remaining 77 fusions were determined based on a common fusion gene (**Supplementary Data 32**).

### 11.g) Confirmation of mutual exclusivity with complete list of oncogenic fusions

With the above curated oncogenic fusion gene pairs and corresponding samples, we re-evaluated the exclusivity rule as described in section **SN 10**. As it turned out, out of 2005 patients with subtype-defining oncogenic fusions, only 7 (0.35%) have ≥2 oncogenic fusions. Among these 7 patients, we discovered that SJBALL020141_D1 and SJBALL020142_D1 (both have oncogenic fusions *MEF2D-DAZAP1* and *KMT2A-MATR3*) were sequenced on the same Illumina instrument (HWI-ST1188) with same flowcell (C49UKACXX) that may can cause contamination and lead to our observation. This mechanism may also apply to another two patients/samples, SJCBF124_D and SJCBF149_D (both have *RUNX1-RUNX1T1* and *CBFB-MYH11*). Another patient PT_7DTGJYA7 was detected fusion *FGFR1-TACC1* from one sample a9a478fc-6897-4c9c-b9f6-ffd2130f5166 and the other fusion *FGFR3-TACC3* from another sample b3a2e094-e0fc-48a9-918a-efac4aa7f2fd. Therefore, we believe there are no more than 2 patients (0.1%) with ≥2 oncogenic fusions out of 2005 fusion positive patients.

When promoter-hijacking were considered, we observed frequent overlap between *HOXA*/*HOXB* cluster and *KMT2A*-rearranged AML that is consistent with previous report[34], as well as overlap between *MYC* enhancer alteration[35] and several oncogenic fusions such as *RUNX1-RUNX1T1* in various cancer types that may worth further functional validation. By excluding *HOX* and *MYC*, we observed an additional 7 patients (out of 2138 fusion positive patients, 0.33%) with double oncogenic fusions, and these 7 patients all have *CRLF2* promoter hijacking, indicating a low but significant potential for *CRLF2* aberrant expression as a nested oncogenic fusion (**Supplementary Data 33)**.

Overall, these data strongly support our application of mutual exclusivity rule to the millions of predictions that enabled systematic manual examination of 5,190 patients (5687 samples) in ~5 hours (of an experienced scientist), with false negative rate <7% (by not reviewing low-vote predictions). We expect this framework will prove valuable for the research community on the analysis of oncogenic fusions.

## 12. Truncating fusion candidates

Although our reasoning in section **SN 8.a** indicates that RNA-seq data is not a good platform to study tumor suppressor genes, here we decided to show a few "recurrent truncating fusions" in response to Reviewer #2's request. The first significant example is *NBAS* truncating fusions. Although interesting at a first glance, a careful examination indicated that *NBAS* is a neighbor gene of *MYCN*, which is frequently amplified in neuroblastoma and Wilms tumor, less frequently in brain tumors or rhabdoid tumors. Indeed, *NBAS*-truncating fusions are mostly found in neuroblastoma (samples with a tag of "SJNBL") and Wilms tumor (samples with a tag of "SJWLM"; **Supplementary Data 21**). We added comments to other truncating candidate fusion on tumor suppressor genes such as *NF1*, *PTEN*, *TP53*, *RB1*, etc. in **Supplementary Data 21** and **Supplementary Data 22**.

## 13. Model of selection bias

Our study indicated that many fusion gene pairs have intronic versioning, where slightly different oncoproteins can be generated in different patient tumors. A natural question is whether such difference may carry biological significance such as oncogenicity. For this, we performed a theoretical analysis (**Supplementary Fig.21**). Here, we introduced the concept of DNA rearrangement events before and after selection. First, DNA rearrangement events can happen either in a random fashion (**Supplementary Fig.21a**) or in a non-random fashion (**Supplementary Fig.21b**). Second, random DNA rearrangement events may be subjected to unbiased (**Supplementary Fig.21c**) or biased **(Supplementary Fig.21d**) selection, which will generate unbiased or biased patient prevalence among different intronic versions, respectively. Clearly, biased DNA rearrangement events (**Supplementary Fig.21b,e**) can confound the statistical analysis and we therefore dropped *TCF3-PBX1* (**Fig. 2l**) and *RUNX1-RUNX1T1* (**Supplementary Fig. 3d**) from this analysis. Fusions with alternative splicing can also confound this analysis and are dropped (**Fig. 5b**).

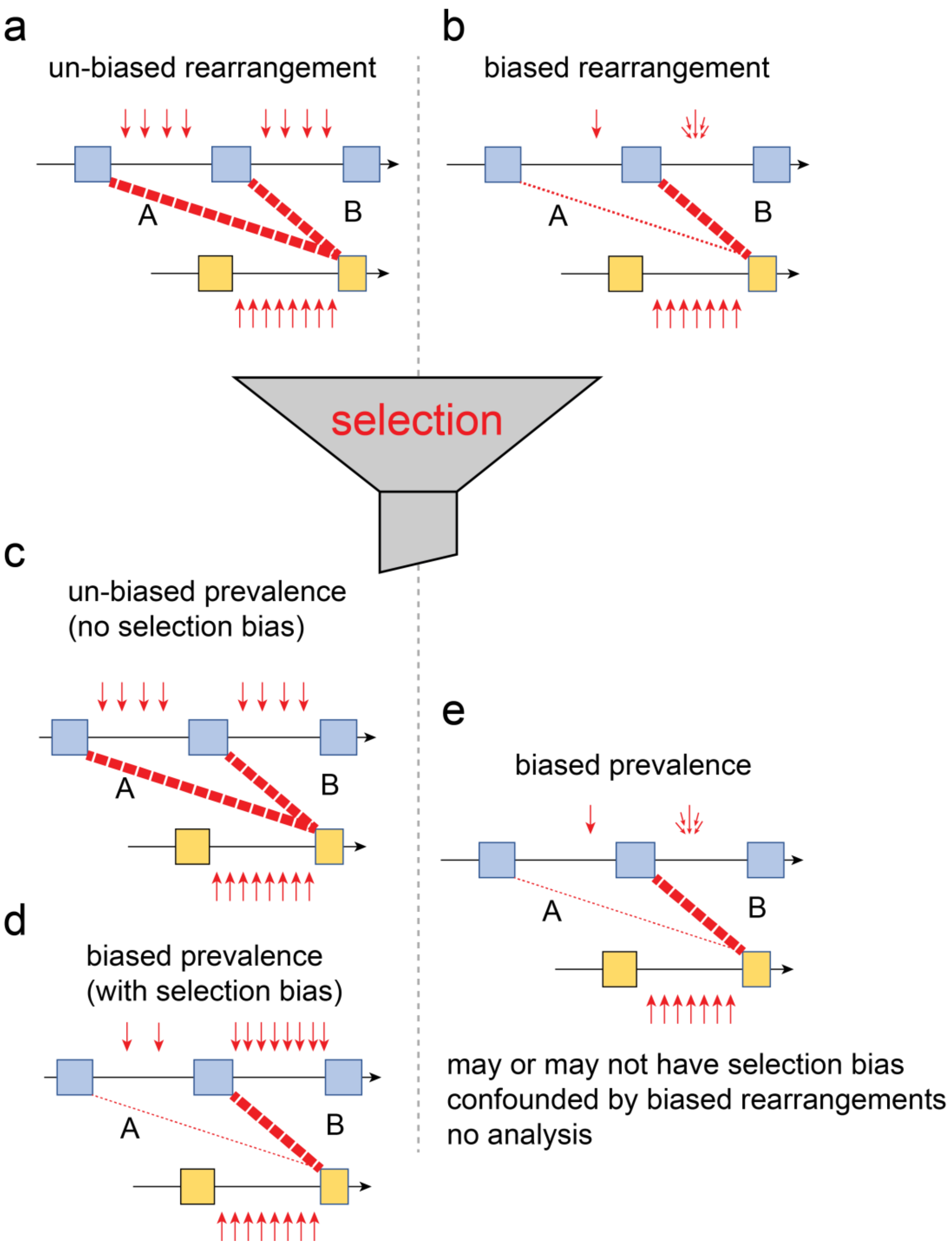


**Supplementary Fig. 21 Selection bias.** DNA rearrangement events can happen in a random fashion (a; such as **Fig. 2j-k**) or in a non-random fashion due to an (unknown, such as the *TCF3-PBX1* example in **Fig. 2l**) molecular mechanism (b). In either scenario, two oncoproteins (A and B) are generated, regardless of the exact DNA breakpoints, due to splicing. Next, selection come to play. First, the oncogenicity of proteins A and B might be same. In this case, selection would be neutral between A and B versions, and the original un-biased rearrangements are mirrored in un-biased patient prevalence (c). Second, the oncogenicity of proteins A and B might be different. In this case, selection bias will be reflected in biased patient prevalence (where version A has less frequency than expected; d). Clearly, selection bias cannot

be analyzed when DNA rearrangement is biased (panels e and b) and when alternative splicing is observed (**Fig. 5b**).


1 Rowley, J. D. Letter: A new consistent chromosomal abnormality in chronic myelogenous leukaemia identified by quinacrine fluorescence and Giemsa staining. *Nature* **243**, 290-293, doi:10.1038/243290a0 (1973).

2 Carroll, A. J. *et al.* Pre-B cell leukemia associated with chromosome translocation 1;19. *Blood* **63**, 721-724 (1984).

3 Hunger, S. P. *et al.* The t(1;19)(q23;p13) results in consistent fusion of E2A and PBX1 coding sequences in acute lymphoblastic leukemias. *Blood* **77**, 687-693 (1991).

4 Raimondi, S. C. *et al.* New recurring chromosomal translocations in childhood acute lymphoblastic leukemia. *Blood* **77**, 2016-2022 (1991).

5 Hunger, S. P., Ohyashiki, K., Toyama, K. & Cleary, M. L. Hlf, a novel hepatic bZIP protein, shows altered DNA-binding properties following fusion to E2A in t(17;19) acute lymphoblastic leukemia. *Genes Dev* **6**, 1608-1620, doi:10.1101/gad.6.9.1608 (1992).

6 Delattre, O. *et al.* Gene fusion with an ETS DNA-binding domain caused by chromosome translocation in human tumours. *Nature* **359**, 162-165, doi:10.1038/359162a0 (1992).

7 Parker, M. *et al.* C11orf95-RELA fusions drive oncogenic NF-kappaB signalling in ependymoma. *Nature* **506**, 451-455, doi:10.1038/nature13109 (2014).

8 Zhang, J. *et al.* Whole-genome sequencing identifies genetic alterations in pediatric low-grade gliomas. *Nat Genet* **45**, 602-612, doi:10.1038/ng.2611 (2013).

9 Ma, X. *et al.* Pan-cancer genome and transcriptome analyses of 1,699 paediatric leukaemias and solid tumours. *Nature* **555**, 371-376, doi:10.1038/nature25795 (2018).

10 Tian, L. *et al.* Long-read sequencing unveils IGH-DUX4 translocation into the silenced IGH allele in B-cell acute lymphoblastic leukemia. *Nature communications* **10**, 2789, doi:10.1038/s41467-019-10637-8 (2019).

11 Liu, Y. *et al.* The genomic landscape of pediatric and young adult T-lineage acute lymphoblastic leukemia. *Nat Genet* **49**, 1211-1218, doi:10.1038/ng.3909 (2017).

12 Northcott, P. A. *et al.* Enhancer hijacking activates GFI1 family oncogenes in medulloblastoma. *Nature* **511**, 428-434, doi:10.1038/nature13379 (2014).

13 Li, B. *et al.* Therapy-induced mutations drive the genomic landscape of relapsed acute lymphoblastic leukemia. *Blood* **135**, 41-55, doi:10.1182/blood.2019002220 (2020).

14 Montefiori, L. E. *et al.* Enhancer Hijacking Drives Oncogenic BCL11B Expression in Lineage-Ambiguous Stem Cell Leukemia. *Cancer Discov* **11**, 2846-2867, doi:10.1158/2159-8290.CD-21-0145 (2021).

15 Mansour, M. R. *et al.* Oncogene regulation. An oncogenic super-enhancer formed through somatic mutation of a noncoding intergenic element. *Science* **346**, 1373-1377, doi:10.1126/science.1259037 (2014).

16 Arabzade, A. *et al.* ZFTA-RELA Dictates Oncogenic Transcriptional Programs to Drive Aggressive Supratentorial Ependymoma. *Cancer Discov* **11**, 2200-2215, doi:10.1158/2159-8290.CD-20-1066 (2021).

17 Druker, B. J. *et al.* Efficacy and safety of a specific inhibitor of the BCR-ABL tyrosine kinase in chronic myeloid leukemia. *N Engl J Med* **344**, 1031-1037, doi:10.1056/NEJM200104053441401 (2001).

18 Ma, X. *et al.* Rise and fall of subclones from diagnosis to relapse in pediatric B-acute lymphoblastic leukaemia. *Nature communications* **6**, 6604, doi:10.1038/ncomms7604 (2015).

19 Tian, L. *et al.* CICERO: a versatile method for detecting complex and diverse driver fusions using cancer RNA sequencing data. *Genome Biol* **21**, 126, doi:10.1186/s13059-020-02043-x (2020).
20 Lawrence, M. S. *et al.* Mutational heterogeneity in cancer and the search for new cancer-associated genes. *Nature* **499**, 214-218, doi:10.1038/nature12213 (2013).
21 Zack, T. I. *et al.* Pan-cancer patterns of somatic copy number alteration. *Nat Genet* **45**, 1134-1140, doi:10.1038/ng.2760 (2013).
22 Grobner, S. N. *et al.* The landscape of genomic alterations across childhood cancers. *Nature* **555**, 321-327, doi:10.1038/nature25480 (2018).
23 Vogelstein, B. *et al.* Cancer genome landscapes. *Science* **339**, 1546-1558, doi:10.1126/science.1235122 (2013).
24 McLeod, C. *et al.* St. Jude Cloud: A Pediatric Cancer Genomic Data-Sharing Ecosystem. *Cancer Discov* **11**, 1082-1099, doi:10.1158/2159-8290.CD-20-1230 (2021).
25 Pui, C. H. & Evans, W. E. Acute lymphoblastic leukemia. *N Engl J Med* **339**, 605-615, doi:10.1056/NEJM199808273390907 (1998).
26 Pugh, T. J. *et al.* The genetic landscape of high-risk neuroblastoma. *Nat Genet* **45**, 279-284, doi:10.1038/ng.2529 (2013).
27 Brady, S. W. *et al.* Pan-neuroblastoma analysis reveals age- and signature-associated driver alterations. *Nature communications* **11**, 5183, doi:10.1038/s41467-020-18987-4 (2020).
28 Versteege, I. *et al.* Truncating mutations of hSNF5/INI1 in aggressive paediatric cancer. *Nature* **394**, 203-206, doi:10.1038/28212 (1998).
29 Zhang, J. *et al.* A novel retinoblastoma therapy from genomic and epigenetic analyses. *Nature* **481**, 329-334, doi:10.1038/nature10733 (2012).
30 Umeda, M. *et al.* Integrated genomic analysis identifies UBTF tandem duplications as a recurrent lesion in pediatric acute myeloid leukemia. *Blood Cancer Discov*, doi:10.1158/2643-3230.BCD-21-0160 (2022).
31 Brady, S. W. *et al.* The genomic landscape of pediatric acute lymphoblastic leukemia. *Nat Genet* **54**, 1376-1389, doi:10.1038/s41588-022-01159-z (2022).
32 Uhrig, S. *et al.* Accurate and efficient detection of gene fusions from RNA sequencing data. *Genome Res* **31**, 448-460, doi:10.1101/gr.257246.119 (2021).
33 Hiwatari, M. *et al.* Molecular studies reveal MLL-MLLT10/AF10 and ARID5B-MLL gene fusions displaced in a case of infantile acute lymphoblastic leukemia with complex karyotype. *Oncol Lett* **14**, 2295-2299, doi:10.3892/ol.2017.6430 (2017).
34 Ayton, P. M. & Cleary, M. L. Transformation of myeloid progenitors by MLL oncoproteins is dependent on Hoxa7 and Hoxa9. *Genes Dev* **17**, 2298-2307, doi:10.1101/gad.1111603 (2003).
35 Zhou, X. *et al.* Exploration of Coding and Non-coding Variants in Cancer Using GenomePaint. *Cancer Cell* **39**, 83-95 e84, doi:10.1016/j.ccell.2020.12.011 (2021).
