# Supplementary material for: Etiology of oncogenic fusions in 5,190 childhood cancers and its clinical and therapeutic implication
Source: Nat Commun. 2023 Apr 5;14:1739. doi: 10.1038/s41467-023-37438-4 (PMC10076316; doi:10.1038/s41467-023-37438-4)
Supplement: Supplementary file 3 — Description to Additional Supplementary Information [file 41467_2023_37438_MOESM3_ESM.pdf]

Description of Additional Supplementary Files

Supplementary Data 1 Samples used in this study.
Supplementary Data 2 Data source of this study.
Supplementary Data 3 Fusions detected in this study.
Supplementary Data 4 Neo splicing events.
Supplementary Data 5 Neo translational events.
Supplementary Data 6 Chimeric exon events.
Supplementary Data 7 Fusion versioning events.
Supplementary Data 8 DNA breakpoints detected from RNAseq data.
Supplementary Data 9 Analysis of expression dominance.
Supplementary Data 10 9525 GTEx Samples used in this work.
Supplementary Data 11 Analysis of alternative splicing.
Supplementary Data 12 Analysis of relative selection bias for intronic versioning
Supplementary Data 13 Fusion isoform detected from 2 typical TCF3-HLF rearrangements.
Supplementary Data 14 Guide RNA and primer sequences.
Supplementary Data 15 Capture sequencing post CRISPR editing.
Supplementary Data 16 CRISPR editing in HAL-01.
Supplementary Data 17 CRISPR editing in UoC-B1.
Supplementary Data 18 Driver gene curation based on known studies.
Supplementary Data 19 Fusion pair list detected in this study based on Supplementary Data 18
Supplementary Data 20 Position specific weight matrix (PWM).
Supplementary Data 21 Manual review of candidate fusions (four methods).
Supplementary Data 22 Manual review of candidate fusions (three methods).
Supplementary Data 23 Manually identified rare oncogenic fusions from Supplementary Data 21-22.
Supplementary Data 24 Newly detected Tier2 fusions from Supplementary Data 21-22.
Supplementary Data 25 Literature support of oncogenic fusions included in this report.
Supplementary Data 26 Promoter hijacking fusions in Arriba output.
Supplementary Data 27 Promoter hijacking fusions in Cicero output.
Supplementary Data 28 Promoter hijacking fusions in FusionCatcher output.
Supplementary Data 29 Promoter hijacking fusions in StarFusion output.
Supplementary Data 30 Harmonization of promoter hijacking fusions.
Supplementary Data 31 Screening of chimeric oncogenic fusions.
Supplementary Data 32 Determination of functional orientation of oncogenic fusions.
Supplementary Data 33 Mutual exclusivity.
