# Supplementary material for: Etiology of oncogenic fusions in 5,190 childhood cancers and its clinical and therapeutic implication
Source: Nat Commun. 2023 Apr 5;14:1739. doi: 10.1038/s41467-023-37438-4 (PMC10076316; doi:10.1038/s41467-023-37438-4)
Supplement: Supplementary file 5 — Reporting Summary [file 41467_2023_37438_MOESM5_ESM.pdf]

nature portfolio

Corresponding author(s): Xiaotu Ma; Shondra Miller; Patrick Brown; Soheil Meshinchi; Jeff Klco

Last updated by author(s): 03/01/2023

# Reporting Summary

Nature Portfolio wishes to improve the reproducibility of the work that we publish. This form provides structure for consistency and transparency in reporting. For further information on Nature Portfolio policies, see our Editorial Policies and the Editorial Policy Checklist.

## Statistics

For all statistical analyses, confirm that the following items are present in the figure legend, table legend, main text, or Methods section.

| n/a | Confirmed | |
|---|---|---|
| ☐ | ☒ | The exact sample size (*n*) for each experimental group/condition, given as a discrete number and unit of measurement |
| ☐ | ☒ | A statement on whether measurements were taken from distinct samples or whether the same sample was measured repeatedly |
| ☐ | ☒ | The statistical test(s) used AND whether they are one- or two-sided<br>*Only common tests should be described solely by name; describe more complex techniques in the Methods section.* |
| ☒ | ☐ | A description of all covariates tested |
| ☐ | ☒ | A description of any assumptions or corrections, such as tests of normality and adjustment for multiple comparisons |
| ☐ | ☒ | A full description of the statistical parameters including central tendency (e.g. means) or other basic estimates (e.g. regression coefficient) AND variation (e.g. standard deviation) or associated estimates of uncertainty (e.g. confidence intervals) |
| ☐ | ☒ | For null hypothesis testing, the test statistic (e.g. *F*, *t*, *r*) with confidence intervals, effect sizes, degrees of freedom and *P* value noted<br>*Give P values as exact values whenever suitable.* |
| ☒ | ☐ | For Bayesian analysis, information on the choice of priors and Markov chain Monte Carlo settings |
| ☒ | ☐ | For hierarchical and complex designs, identification of the appropriate level for tests and full reporting of outcomes |
| ☐ | ☒ | Estimates of effect sizes (e.g. Cohen's *d*, Pearson's *r*), indicating how they were calculated |

*Our web collection on statistics for biologists contains articles on many of the points above.*

## Software and code

Policy information about availability of computer code

| | |
|---|---|
| Data collection | no software used |
| Data analysis | Arriba (v1.2.0); Cicero (v0.3.0); STAR-Fusion (v1.6.0); FusionCatcher (v1.33); FusionCatcher (v1.10); STAR v2.5.3a; All in-house scripts for this work are deposited in zenodo (https://doi.org/10.5281/zenodo.7510612) |

For manuscripts utilizing custom algorithms or software that are central to the research but not yet described in published literature, software must be made available to editors and reviewers. We strongly encourage code deposition in a community repository (e.g. GitHub). See the Nature Portfolio guidelines for submitting code & software for further information.

## Data

Policy information about availability of data

All manuscripts must include a data availability statement. This statement should provide the following information, where applicable:

- Accession codes, unique identifiers, or web links for publicly available datasets
- A description of any restrictions on data availability
- For clinical datasets or third party data, please ensure that the statement adheres to our policy

All genomics datasets used for this work are from public resources detailed in Supplementary Table 2, including CBTN [https://portal.kidsfirstdrc.org], FredHutch [https://portal.gdc.cancer.gov], TARGET [https://ocg.cancer.gov/programs/target/data-matrix], G4K, PCGP, RTGC [https://platform.stjude.cloud/data/cohorts], Rhabdomyosarcoma (PMID:24436047) [https://www.ncbi.nlm.nih.gov/projects/gap/cgi-bin/study.cgi?study_id=phs000720.v4.p1], Ewing Sarcoma

(PMID:25010205) [https://www.ncbi.nlm.nih.gov/projects/gap/cgi-bin/study.cgi?study_id=phs000768.v2.p1 and https://www.ncbi.nlm.nih.gov/projects/gap/cgi-bin/study.cgi?study_id=phs000804.v1.p1], AML (PMID:27798625) [https://platform.stjude.cloud/data/cohorts, SJC-DS-1013], MDS (PMID:29146900) [https://ega-archive.org/datasets/EGAD00001003782], AML (PMID:30760869) [https://ocg.cancer.gov/programs/target/data-matrix], Erythroleukemia (PMID:30926971) [https://ega-archive.org/studies/EGAS00001002537], AML (PMID:31350825) [https://platform.stjude.cloud/data/cohorts], B-ALL (PMID:31697823) [https://ngdc.cncb.ac.cn/gsa-human/browse/HRA000119], tMN (PMID:33579957) [https://ega-archive.org/datasets/EGAD00001006674], AML (PMID:34778799) [https://ega-archive.org/datasets/EGAD00001006444], AML (PMID:35176137) [https://ega-archive.org/datasets/EGAD00001008407]. The total RNAseq data and WGS data generated in this study for HAL-01 and UoC-B1 cell lines are deposited at ENA [https://www.ebi.ac.uk/ena/browser/view/PRJEB55308]. The time series data post CRISPR editing for cell line HAL-01 and UoC-B1 is deposited in zenodo [https://doi.org/10.5281/zenodo.7510612]. All data generated in this study are provided in Source Data file. All input data and plot scripts for figures can also be found in zenodo [https://doi.org/10.5281/zenodo.7510612]. All these data are under restricted access and an Institutional Review Board (IRB) approval was obtained to request access to these data.

## Human research participants

Policy information about studies involving human research participants and Sex and Gender in Research.

| | |
|---|---|
| Reporting on sex and gender | Sex and gender were not considered in the study design and samples were analyzed based on availability in publicly available resources. |
| Population characteristics | Not Applicable |
| Recruitment | Not Applicable |
| Ethics oversight | Not Applicable |

Note that full information on the approval of the study protocol must also be provided in the manuscript.

# Field-specific reporting

Please select the one below that is the best fit for your research. If you are not sure, read the appropriate sections before making your selection.

☒ Life sciences ☐ Behavioural & social sciences ☐ Ecological, evolutionary & environmental sciences

For a reference copy of the document with all sections, see nature.com/documents/nr-reporting-summary-flat.pdf

# Life sciences study design

All studies must disclose on these points even when the disclosure is negative.

| | |
|---|---|
| Sample size | Not Applicable |
| Data exclusions | Not Applicable |
| Replication | Not Applicable |
| Randomization | Not Applicable |
| Blinding | Not Applicable |

# Reporting for specific materials, systems and methods

We require information from authors about some types of materials, experimental systems and methods used in many studies. Here, indicate whether each material, system or method listed is relevant to your study. If you are not sure if a list item applies to your research, read the appropriate section before selecting a response.

## Materials & experimental systems

| n/a | Involved in the study | |
|---|---|---|
| ☒ | ☐ | Antibodies |
| ☐ | ☒ | Eukaryotic cell lines |
| ☒ | ☐ | Palaeontology and archaeology |
| ☒ | ☐ | Animals and other organisms |
| ☒ | ☐ | Clinical data |
| ☒ | ☐ | Dual use research of concern |

## Methods

| n/a | Involved in the study | |
|---|---|---|
| ☒ | ☐ | ChIP-seq |
| ☒ | ☐ | Flow cytometry |
| ☒ | ☐ | MRI-based neuroimaging |

## Eukaryotic cell lines

Policy information about cell lines and Sex and Gender in Research

| | |
|---|---|
| Cell line source(s) | Cell line HAL-01 was purchased from DSMZ (catalog #ACC 610). UoC-B1 was from Yang lab (Jun J. Yang) in St. Jude Childrens Research Hospital. |
| Authentication | For both cell lines, STR profiling, whole genome and transcriptome sequencing were performed to confirm identify and DNA and RNA breakpoints. |
| Mycoplasma contamination | Both cell lines are negative for mycoplasma contamination using MycoAlert Mycoplasma Detection Kit (Lonza). |
| Commonly misidentified lines (See ICLAC register) | No. The 2 cell lines used in this study are not in the misidentified lines list. |
